# The Effects of Exercise-Based Interventions on Fluid Overload Symptoms in Patients with Heart Failure: A Systematic Review and Meta-Analysis

**DOI:** 10.3390/biomedicines10051111

**Published:** 2022-05-11

**Authors:** Mei Rosemary Fu, Yuan Li, Catherine Conway, Alessandra Masone, Jinbo Fang, Christopher Lee

**Affiliations:** 1 School of Nursing–Camden, Rutgers, The State University of New Jersey, Camden, NJ 08102, USA; 2 West China School of Nursing, Sichuan University, Chengdu 610041, China; lynetteli1992@stu.scu.edu.cn (Y.L.); fangjinbo1107@163.com (J.F.); 3 Key Laboratory of Birth Defects and Related Diseases of Women and Children, Sichuan University, Ministry of Education, Chengdu 610041, China; 4 William F. Connell School of Nursing, Boston College, Chestnut Hill, MA 02467, USA; conwayck@bc.edu (C.C.); masoneal@bc.edu (A.M.); christopher.lee.8@bc.edu (C.L.)

**Keywords:** heart failure, exercise, nursing, fluid overload, symptom, dyspnea, fatigue, systematic review

## Abstract

Patients with heart failure are subjected to a substantial burden related to fluid overload symptoms. Exercise can help the lymphatic system function more effectively to prevent fluid build-up in tissues and interstitium, thus potentially mitigating the symptoms due to fluid overload. The objective of this systematic review was to examine the effects of exercise-based interventions on fluid overload symptoms among patients with heart failure. MEDLINE, Embase, Cochrane Library, and CINAHL databases were systematically searched for relevant studies published from inception to August 2021. We included randomized controlled trials that compared exercise-based interventions of different modalities and usual medical care for adult patients with heart failure and reported the effects of interventions on any symptoms related to fluid overload. A random-effects meta-analysis was used to estimate the effectiveness, and a subgroup analysis and univariate meta-regression analysis were used to explore heterogeneity. Seventeen studies covering 1086 participants were included. We found robust evidence indicating the positive effect of exercises in dyspnea relief (SMD = −0.48; 95%CI [−0.76, −0.19]; *p* = 0.001); the intervention length also influenced the treatment effect (β = 0.033; 95%CI [0.003, 0.063]; *p* = 0.04). Initial evidence from existing limited research showed that exercise-based intervention had positive effect to alleviate edema, yet more studies are needed to verify the effect. In contrast, the exercise-based interventions did not improve fatigue compared with usual care (SMD = −0.27; 95%CI [−0.61, 0.06]; *p* = 0.11). Findings regarding the effects of exercises on bodily pain, gastro-intestinal symptoms, and peripheral circulatory symptoms were inconclusive due to limited available studies. In conclusion, exercise-based interventions can be considered as an effective nonpharmacological therapy for patients with heart failure to promote lymph flow and manage fluid overload symptoms. Exercise-based interventions seem to have very limited effect on fatigue. More research should investigate the mechanism of fatigue related to heart failure. Future studies with high methodological quality and comprehensive assessment of symptoms and objective measure of fluid overload are warranted.

## 1. Introduction

Heart failure (HF) is a growing public health problem affecting 26 million people worldwide [1]. Fluid overload is the hallmark of HF that leads to hemodynamic and clinical congestion [2]. The accumulation of body fluids progressively manifests itself in multiple congestion symptoms, causing patient distress [3]. Fluid overload symptoms in patients with HF can be defined as symptoms that impede multiple body system functions due to fluid retention or redistribution and can be categorized into symptoms of pulmonary congestion (e.g., dyspnea, orthopnea, coughing, or wheezing) and systemic venous congestion (e.g., edema, fatigue, bodily pain, and gastrointestinal disturbance) [4,5]. The occurrence or exacerbation of fluid overload symptoms is the major reason for patients with HF to be admitted or readmitted to hospital, imposing considerable burdens on the individual, family, and health care system [2,3,6].

The surveillance and management of worsening fluid overload symptoms is the cornerstone of HF care [7]. Diuretics are the mainstay of therapy to relieve congestive or fluid overload symptoms, but often not effective in patients with diuretic resistance or renal insufficiency [8]. Ultrafiltration may be considered as an alternative method for patients with refractory congestion not responding to medical therapy [9], but the associated cost, the need for veno-venous access, provider experience, and nursing support remain concerns about the routine use of this technique [10]. Dietary sodium and water restriction enhance volume management with diuretics; however, the widely embraced dictum of sodium restriction is not well supported and fluid restriction is also difficult to achieve and maintain [10,11]. Recent research on the role of lymphatic system in the pathogenesis of cardiovascular diseases and advances in this field have highlighted new therapeutic perspectives of fluid volume removal or fluid overload management in HF [12,13,14].

In normal circulation, there is continuous filtration of fluid, along with some proteins, from the intravascular space into the tissues at a rate dependent on the gradient between the intravascular and extravascular hydrostatic pressure. The lymphatic vasculature running in parallel to the blood venous system has an essential role in maintaining interstitial milieu by draining filtered fluid to the venous circulation [15,16]. It is estimated that up to eight liters per day of interstitial fluid are collected by the initial lymphatics that line all organs and are drained by the unidirectional lymphatics to the blood circulatory system [15]. The initial lymphatics have endothelial microvalves and classical intralymphatic valves to provide a mechanism for unidirectional flow during compression and expansion of the initial lymphatics [17]. These compression and expansion movements depend on muscle contraction, breathing movements (particularly inspiration), arterial pulsations, postural changes, and skin tension [17]. If fluid is not drained at the same rate as it leaks, the painful and debilitating condition of fluid overload would develop [15,16]. Thus, interventions focusing on optimizing lymph flow is a potential therapeutic approach to management of fluid overload symptoms in HF.

Exercise that stimulates muscle contractions and breathing can help the lymphatic system function more effectively and potentially prevent or decrease fluid accumulation in tissues and interstitium [17,18]. The effect of exercise on lymph formation and transport has been directly estimated with intact lymphatics in anaesthetized sheep using cannulation of a lymphatic duct [19,20]. In response to prolonged exercise (2-h steady-state exercise at 70% maximum heart rate) in sheep, the lymphatic propulsion rate immediately accelerated and lymph flow increased five-fold from resting values during the first 15 min, and then gradually decreased to a constant 130% above baseline during the last 30 min of exercise [20]. Havas et al. [21] demonstrated lymph flow dynamics in exercising human skeletal muscle using lymphoscintigraphy. The results showed that the lymph clearance rate was low during the rest periods, though higher in the endurance-trained than in the sedentary subjects, and the clearance rate was elevated three- to six-fold during exercise compared with resting levels [21].

Therefore, we hypothesize that exercise-based interventions that produce propulsive and centripetal movement of lymph could accelerate the trafficking of body fluids through lymphatic vessels to promote fluid draining and, thus, alleviate the symptoms due to fluid overload in patients with HF. The purpose of the systematic review and meta-analysis was to comprehensively synthesize and evaluate the effectiveness of exercise-based interventions on fluid overload symptoms in patients with HF. The specific review questions were: (1) what are the effects of exercise-based interventions on fluid overload symptoms in patients with HF? (2) Which type of exercise is superior in achieving positive outcomes among the population? (3) Is there any trial-level characteristics potentially affecting the overall effectiveness?

## 2. Materials and Methods

This was a systematic review and meta-analysis of randomized controlled trials (RCTs) that compared the effects of exercise-based interventions on fluid overload symptoms with usual care among adult people with HF. This review work was prospectively registered with the PROSPERO database (CRD42021260235) and is reported in accordance with the PRISMA 2020 statement [22].

### 2.1. Search Strategy

We systematically searched the MEDLINE, Embase, Cochrane Library, and CINAHL databases from inception to 3 August 2021, to identify relevant literature with no restrictions on language or year of publication. We also manually searched the bibliographies of all relevant articles for additional eligible studies. The search strategy was devised using a combination of Medical Subject Headings (MeSH) and free text terms with synonyms that encapsulated heart failure, exercise, fluid overload, symptoms, and randomized clinical trials. Complete search syntax for each database can be found in Supplementary Chart S1: Search strategy.

### 2.2. Eligibility Criteria

In this systematic review, we only considered RCTs that satisfied the following inclusion criteria: (1) enrolled patients aged 18 years or older with stable HF; (2) compared exercise-based interventions of different modalities versus usual medical care that did not include structured exercise training but may include some form of active intervention (e.g., education, psychological intervention, etc.); (3) reported the effects of interventions on any of fluid overload symptoms; and (4) followed up participants for at least 4 weeks. We excluded studies that (1) addressed participants with general cardiac disorders other than HF specifically or participants with deteriorated HF requiring hospitalization; (2) failed to provide adequate descriptions for the intervention; or (3) measured fluid overload symptoms using unvalidated instruments.

### 2.3. Study Selection, Data Extraction, and Quality Appraisal

After removing duplicate articles, Y.L., C.C., and A.M. independently screened all titles and abstracts for potential relevance. Full texts were retrieved for articles deemed potentially relevant and reviewed against the eligibility criteria for inclusion. A standardized data abstraction table [23,24] was compiled and piloted to extract data of interest from the included studies for assessment of study quality and evidence synthesis. Extracted data items included author(s), year of publication, country of origin, setting and sample, patient characteristics, intervention and comparator details (e.g., specific exercise contents, frequency, duration, intensity, etc.), relevant outcome(s), adverse events, and information concerning the studies’ methodology quality. Two review authors (C.C. and A.M.) completed data extraction and cross-checked the extracted information. The co-first authors (M.R.F. and Y.L.) then rechecked all the extracted results from each included study and finalized the data abstraction table.

The quality of the included RCTs was assessed independently by two authors (C.C. and A.M.) using the updated Cochrane risk-of-bias tool for randomized trials (RoB 2) [25] and verified by the co-first authors (M.R.F. and Y.L.). The quality of the included RCTs was assessed in terms of risk of bias in five bias domains including randomization process, deviation from intended intervention, missing outcome data, measurement of the outcome, and selection of reported results. Response options of “low risk of bias”, “some concerns”, or “high risk of bias” within each domain allow for reaching an overall risk-of-bias judgment for the result being assessed and, thus, enabling stratification of studies by the overall judgment as a meta-analysis strategy [25]. Divergent options were resolved by discussion among the authors until consensus was reached.

### 2.4. Data Synthesis

Statistical analyses were done with RevMan version 5.4 (the Cochrane Collaboration, 2020, London, United Kingdom; https://training.cochrane.org/online-learning/core-software/revman, accessed on 22 April 2022) and STATA 15.1 (Stata Corp, College Station, TX, USA). Eligible studies were analyzed based on change-from-baseline measures for fluid overload symptoms over the intervention period and studies that only provided the mean value at endpoint will be otherwise summarized descriptively. The mean change in each group was obtained, if necessary, by subtracting the baseline mean from the post-intervention mean and the standard deviations for changes were imputed according to the formula provided by Cochrane [26]. For studies that reported more than one follow-up time point, the first time point after the completion of intervention was selected to calculate the change scores. Meta-analyses were performed using an inverse variance (IV) random-effects model to calculate summary estimates of weighted mean differences (WMDs), or when the outcome was measured by different psychometric instruments, the standardized mean differences (SMDs), along with their respective 95% confidence intervals (CIs) [26]. The random-effects model was preferred where possible for quantitative syntheses due to the anticipated variability in patient populations, exercise interventions, and study settings across the included studies. Between-study heterogeneity was assessed by inspecting the forest plots and calculating the *I*^2^ statistics, whereby an *I*^2^ value ≥ 75% represents considerable heterogeneity [26]. Sensitivity analysis was conducted to test the robustness of the results by removing outlying studies and omitting a single study in turn. For outcomes that were reported by at least 10 studies, we had planned to explore publication bias by visual inspection for funnel plot asymmetry.

Subgroup analysis was conducted by stratifying the included interventions according to the corresponding muscle group contracted during the exercises. Such categorization of the interventions might enable the subgroup analyses to answer which type of exercise is superior in achieving positive outcomes of alleviating fluid overload symptoms among adult people with HF. For particular outcomes with usable data from at least 6 studies [27], exploratory meta-regression was performed to identify whether any trial-level covariate could possibly modify the treatment effects and help explain the heterogeneity. The potential effect modifiers in meta regression were predetermined and consisted of participant characteristics (mean age, male percentage, body mass index, left ventricular ejection fraction, and New York Heart Association), intervention characteristics (intervention length, intervention dose, exercise sessions under supervision or not, and intervention environment), and trial characteristic (overall risk-of-bias judgment).

## 3. Results

### 3.1. Study Selection

**Figure 1:**
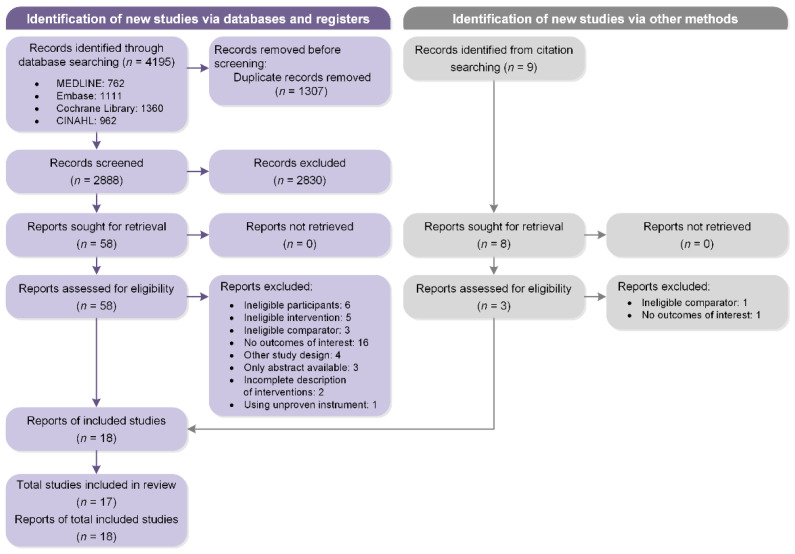
Flow diagram of the study selection process according to PRISMA guidelines.

Figure 1 illustrates our literature search and screening process. The database searching yielded a total of 4195 records. After removal of duplicates, three authors (Y.L., C.C., and A.M.) independently screened all titles and abstracts of the remaining 2888 records and 2830 were excluded because of nonconformity to the eligibility criteria. Full texts of 58 potentially relevant reports were retrieved for detailed adjudication, and then 40 reports were further excluded. With one additional report identified from citation search, 17 studies described in 18 reports were ultimately selected for the current systematic review.

### 3.2. Study Characteristics

The basic information of the included studies is outlined in Table 1 (detailed study characteristics are presented in the Supplementary Table S1 Characteristics of the included studies). All the studies employed an individual-level randomized design. The majority (13/17) of the studies took place in countries or territories classified as the World Bank-defined high-income economies [28], including the United States (7/13), Sweden (1/13), Israel (1/13), Poland (1/13), Korea (1/13), Hong Kong SAR, China (1/13), and Taiwan, China (1/13). The included studies randomized 1086 HF patients with a mean age ranging between 49.4 [29] and 76.2 years [30], and the proportion of females ranging between 0% [31] and 60.7% [32]. Only seven studies specified the baseline mean Body Mass Index (BMI) of participants, which ranged from 25.7 [33] to 34.9 kg/m^2^ [34]. Thirteen studies reported the left ventricular ejection fraction, among which the majority (12/13) recruited participants with a reduced mean value of <40%. Twelve studies measured and reported the severity of HF symptoms with New York Heart Association (NYHA) functional class.

We identified three distinct types of exercise interventions according to the corresponding muscle group contracted during the exercise: respiratory muscle contracted exercises (5/17), peripheral muscle contracted exercises (11/17), and both respiratory and peripheral muscle contracted exercises (1/17). Five studies [32,33,35,36,37] tested breathing exercises or inspiratory muscle training that involved in respiratory muscles or “breathing pump muscles” (i.e., diaphragm and intercostal muscles) contraction. Eleven studies [29,30,31,34,38,39,40,41,42,43,44] involved in peripheral muscle contraction, that is, exercise interventions selectively targeted at modulation of the peripheral skeletal muscles. In this review, peripheral muscle contraction exercises included walking, bicycling, stretching, flexing, peripheral resistance training, muscle tensing and relaxing training, or any combination of those forms of exercises. One study [45] examined the effects of Baduanjin exercise which comprised a set of body movements along with peripheral musculoskeletal stretching coordinated with deep breathing. The frequency of exercise was mainly three sessions per week and the duration of programs ranged from 1 [40] to 18 [34] months. Most of the exercise sessions were under full or partial supervision, in forms of face-to- face or telemonitoring. In addition, a relatively high attrition rate was noted in some of the studies included in this meta-analysis (Table 1).

**Table 1:** Basic information of included studies.

Author, Year (Country)	Allocation	Mean Age, Years	Female, %	Mean BMI, kg/m^2^	Mean LVEF, %	NYHA III-IV, %	Muscle Contracted ^†^	Intervention Duration and Frequency	Supervised ^‡^	Setting	Follow-Ups	Outcomes (Measures)
Alkan, et al., 2017 (Turkey) [32]	IG: 35 CG: 35	64.4 (12.8)	60.7%	NR	NR	73.2%	Respiratory	30 min daily for 3 months	No	Home	Baseline and 3 months	1. Dyspnea (BDI)
Beniaminovitz, et al., 2002 (United States) [29]	IG: 20 CG: 9	49.36 (3.4)	28.0%	NR	19.4 (1.4)	NR	Peripheral	30 min per session, 3 sessions per week for 3 months	Yes	Hospital	3 months	1. Dyspnea (TDI)
Bosnak-Guclu, et al., 2011 (Turkey) [35]	IG: 18 CG:18	67.7 (9.3)	20%	25.9 (3.8)	34.9 (7. 4)	33.3%	Respiratory	30 min daily for 6 weeks	Yes	Home and Rehabilitation center	Baseline and 1.5 months	1. Dyspnea (MMRC); 2. Fatigue (Turkish FSS); 3. Pain (SF-36 bodily pain dimension)
Chen, et al., 2018 (Taiwan, China) [45]	IG: 39 CG:41	70.3 (13.5)	47.5%	NR	58.6 (15.6)	NR	Respiratory and Peripheral	35 min per session, 3 sessions per week for 3 months	No	Home	Baseline, 1 2, and 3 months	1. Fatigue (Chinese PFS)
Corvera-Tindel, et al., 2004 (United States) [39]	IG: 42 CG: 37	62.6 (10.6)	1.3%	29.5 (6.3)	27 (8.8)	19%	Peripheral	10–60 min per session, 5 sessions per week for 3 months	No	Home	3 months	1. Dyspnea (DFI)
Hossein Pour, et al., 2020 (Iran) [33]	IG: 49 CG: 49	56.6 (9.2)	47.6%	25.7 (4.7)	33.1 (5.3)	61.9%	Respiratory	30 min daily for 6 weeks	Yes	Home and rehabilitation center	Baseline and 1.5 months	1. Dyspnea (MMRC); 2. Fatigue (FSS)
Jena, et al., 2020 (India) [40]	IG: 20 CG: 20	NR	37.5%	NR	NR	NR	Peripheral	30 min daily for 1 month	Yes	Home and hospital	1 month	1. Edema (Edema grading scale) 2. Pain (Numeric pain rating scale)
Jin and Lee, 2016 (Korea) [41]	IG: 32 CG: 28	58 (12)	26.8%	NR	31.2 (6.9)	12.2%	Peripheral	50 min per session, 5 sessions per week for 3 months	Yes	Home	Baseline and 3 months	1. Dyspnea (DFI)
Klocek, et al., 2005 (Poland) [31]	IG (A): 14 IG (B): 14 CG: 14	55.9 (8.1)	0%	27 (3.9)	33.2 (3.8)	57.1%	Peripheral	25 min per session, 3 sessions per week for 6 months	Yes	Rehabilitation center	Baseline and 6 months	1. Subjective symptoms (SSA-P)
Norman, et al., 2020 (United States) [34]	IG: 102 CG: 102	60.4 (11.5)	44.6%	34.9 (8.2)	39.9 (13.1)	36.8%	Peripheral	150 min per week for 18 months	Yes	Rehabilitation center	Baseline, 6, 12 and 18 months	1. Fatigue (PROMIS-29)
Pozehl, et al., 2008 (United States) [42]	IG: 16 CG: 7	66.2 (10.2)	9.5%	26.9 (5.6)	28.4 (7.3)	61.9%	Peripheral	60 min per session, 3 sessions per week for 6 months	Yes	Rehabilitation center	Baseline, 3 and 6 months	1. Dyspnea (Dyspnea Index) 2. Fatigue (PFS)
Pozehl, et al., 2010 (United States) [43]	IG: 22 CG: 20	59.9 (13.8)	45.5%	NR	32.7 (6.1)	45.2%	Peripheral	60 min per session, 3 sessions per week for 6 months	Yes	Rehabilitation center	Baseline and 3 months	1. Dyspnea (DFI)
Seo, et al., 2016 (United States) [36]	IG: 18 CG: 18	65.9 (12.4)	28.6%	31.1 (6.3)	37.1 (18.6)	52.3%	Respiratory	15 min per session, two sessions daily, at least 5 days per week for 2 months	No	Home	Baseline, 2 and 5 months	1. Dyspnea (Items from KCCQ; Dyspnea with ADL; Dyspnea with physical functioning)
Wall, et al., 2010 (United States) [44]	IG: 9 CG: 10	69.7 (4.1)	42.1%	NR	NR	NR	Peripheral	15 min per session, 3 sessions per week for 12 months	Yes	Home	Baseline, 6 and 12 months	1. Dyspnea (CHQ Subscale); 2. Fatigue (CHQ Subscale)
Weiner, et al., 1999 (Israel) [37]	IG: 10 CG: 10	65 (4.4)	10%	NR	23.8 (2.2)	NR	Respiratory	60 min daily, 6 times per week for 3 months	Yes	Hospital	Baseline and 3 months	1. Dyspnea (Dyspnea Index)
Willenheimer, et al., 1998 and 2001 Sweden) [38,46]	IG: 23 CG: 27	64 (7.4)	29%	NR	35.6 (10.9)	49.0%	Peripheral	45 min per session, three sessions per week for 4 months	Yes	Rehabilitation center	Baseline, 4 and 10 months	1. Dyspnea (DFI)
Yu, et al., 2007 (Hong Kong, China) [30]	IG: 79 CG: 79	76.2 (7.8)	50.4%	NR	NR	37.2%	Peripheral	60 min per session, 2 sessions per week for 14 weeks	No	Home and hospital	Baseline, 2 and 3.5 months	1. Dyspnea (CHQ Subscale); 2. Fatigue (CHQ Subscale)

Note: ^†^ Muscle contracted categorized according to the peripheral muscles versus respiratory muscles contracted during the exercise-based interventions. ^‡^ Supervised categorized according to the exercise sessions under constant supervision or partial supervision (supervised: yes) versus those not under any supervision (supervised: no). Abbreviations: BMI, Body Mass Index; LVEF, Left Ventricular Ejection Fraction; NYHA, New York Heart Association; NR, not reported; BDI, the Basal Dyspnea Index; TDI, the Transitional Dyspnea Index; MMRC, Modified Medical Research Council dyspnea scale; PFS, Piper Fatigue Scale; FSS, Fatigue Severity Scale; DFI, the Dyspnea Fatigue Index; CHQ, the Chronic Heart Failure Questionnaire; SSA-P, the Subjective Symptoms Assessment Profile; PROMIS-29, patient-reported outcome measurement information systems; KCCQ, Kansas City Cardiomyopathy Questionnaire; IG: Intervention Group; CG: Control Group; ADL: Activities of daily living.

### 3.3. Risk of Bias in Studies

**Figure 2:**
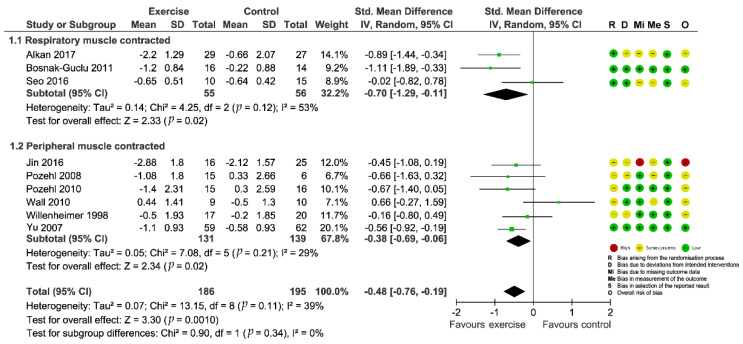
Forest plot showing the effect of exercise-based interventions on dyspnea and risk of bias assessment for each study. (Studies were stratified by the corresponding muscle group contracted during the exercise).

**Figure 3:**
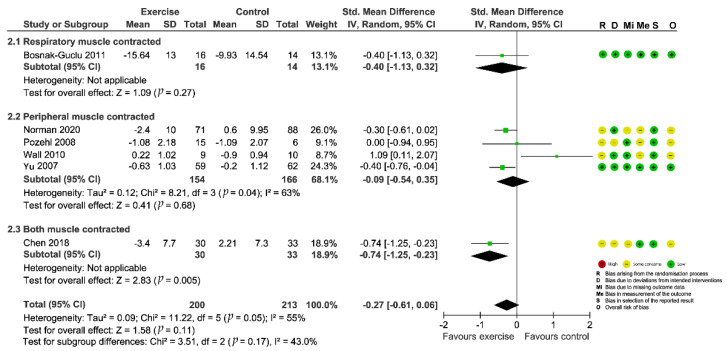
Forest plot showing the effect of exercise-based interventions on fatigue and risk of bias assessment for each study. (Studies are stratified by the corresponding muscle group contracted during the exercise).

The results of risk-of-bias assessment were visualized using the Risk-Of-Bias VISualization tool [47] and embedded in the forest plots (Figure 2 and Figure 3). Systematic biases of the included studies were mainly sourced from the following aspects: (1) 70% (12/17) of studies were considered as “some concerns” in randomization process due to inadequately describing the methods of random-sequence generation and/or allocation concealment; (2) 47% (8/17) of studies were found to have “some concerns” in deviations from the intended interventions as participants were aware of their assigned intervention; (3) 47% (8/17) of studies were considered as “some concerns” and 5% (1/17) were “high risk of bias” in missing outcome data due to their comparatively high attrition rates and a lack of evidence indicating their result were not biased by missing outcome data; and (4) 58% (10/17) of studies were concerned in measurement of the outcome since they did not clearly describe the method for blinding of outcome assessment [25].

### 3.4. Intervention Effect on Dyspnea

**Figure 4:**
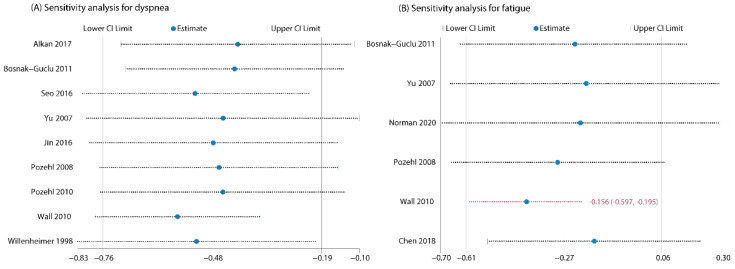
(**A**) Sensitivity analysis for dyspnea by omitting one study in turn (did not change the results of primary meta-analysis); (**B**) Sensitivity analysis for fatigue by omitting one study in turn (changed the results of primary meta-analysis after removing Wall 2010).

A total of 13 studies examined the intervention effect on fluid overload symptoms of dyspnea. Eleven studies [29,30,32,33,35,36,38,41,42,43,44] reported complete data on changes in dyspnea from pre- to post-intervention compared with outcomes from a no-exercise control group, while Corvera-Tindel et al. (2004) [39] only provided endpoint data on dyspnea and Weiner et al. (1999) [37] just offered data for the intervention group. Various psychometric tools were used for evaluation (Basal Dyspnea Index, BDI; Transitional Dyspnea Index, TDI; Modified Medical Research Council dyspnea scale, MMRC; Dyspnea-Fatigue Index, DFI; Dyspnea subscale of Chronic Heart Failure Questionnaire, CHQ subscale; Dyspnea Index; Dyspnea items from Kansas City Cardiomyopathy Questionnaire, KCCQ). We preliminarily combined data from the 11 studies and the results indicated a significant effect (SMD = −1.36; 95%CI [−2.22, −0.50]; *p* = 0.002) in favor of the intervention, however, two outlying studies [29,33] contributed to a considerable heterogeneity (*I*^2^ = 94%; Supplementary Figure S1: Preliminary meta-analysis for dyspnea). So, we excluded the studies from the quantitative analysis. The four studies that did not undergo quantitative analyses because of incomplete data or outlying results consistently concluded that dyspnea was significantly improved in patients who received exercise-based interventions compared with the control group. Finally, we pooled data from nine included trials for the outcome and the aggregated result also showed a significant improvement in dyspnea (SMD = −0.48; 95%CI [−0.76, −0.19]; *p* = 0.001) with acceptable heterogeneity (*I*^2^ = 39%; Figure 2). Sensitivity analyses by sequentially removing each study and reanalyzing the remaining dataset did not trigger major changes in the direction or magnitude of the main finding (Figure 4), thus confirming the robustness of the estimated effect.

Subgroup analyses did not show a significant difference between pooled estimates by exercise types stratified according to the muscle group contracted during the exercise (*p* = 0.34, Figure 2). From the univariate meta-regression, we found the intervention length could significantly influence the overall effect size (β = 0.033; 95%CI [0.003, 0.063]; *p* = 0.04) and help explain a substantial proportion (R^2^ = 43%) of the between-study variance (Table 2). Specifically, the longer the intervention lasted, the higher the positive dyspnea score which represented more severe symptoms. This suggested that the positive impact of exercise on dyspnea might be gradually weakened.

### 3.5. Intervention Effect on Edema

Only one included study [40] evaluated the effect on fluid overload symptom of edema measured by the Edema Grading Scale. The study adopted a post-test-only control group design and suggested that aerobic exercises were highly effective to reduce edema among HF patients receiving conservative treatment (*p* = 0.01).

### 3.6. Intervention Effect on Fatigue

Seven studies [30,33,34,35,42,44,45] evaluated the intervention effect of exercises on fatigue and all the studies reported adequate data for meta-analysis. Different psychometric tools were used for evaluation of fatigue (Fatigue Severity Scale, FSS; Piper Fatigue Scale, PFS; Fatigue subscale of Chronic Heart Failure Questionnaire, CHQ subscale; Patient-Reported Outcome Measurement Information Systems, PROMIS-29). The combined results for the seven RCTs showed that the effect of exercise interventions on fatigue failed to reach statistical significance (SMD = −0.59; 95%CI [−1.33, 0.15]; *p* = 0.12) and the heterogeneity was prohibitively high (*I*^2^ = 92%; Supplementary Figure S2: Preliminary meta-analysis for fatigue). Sensitivity analysis by removing the outlying study [33] greatly decreased the between-study heterogeneity to 51%, but did not change the nonsignificant finding (SMD = −0.27; 95%CI [−0.61, 0.06]; *p* = 0.11; Figure 3). However, when we further removed studies one by one, the summary estimates turned to significant after Wall et al. (2010) [44] was excluded (Figure 4), indicating the results were instable.

Subgroup analyses of included studies found no significant differences between groups based on the muscle group contracted during exercises (*p* = 0.17, Figure 3). Univariate meta-regression on trial-level covariates did not unveil any prespecified covariates that could modify the overall effect estimate (Table 2). These characteristics accordingly might not help explain the heterogeneity observed across studies, nor did they affect the overall effectiveness.

### 3.7. Intervention Effect on Other Fluid Overload Symptoms

Two studies [35,40] reported data on bodily pain, among which Bosnak-Guclu et al. (2011) [35] used the bodily pain subscale of SF-36 for measurement and Jena et al. (2020) [40] did not specify the measuring instrument. The limited number of studies prevented a pooled analysis of the results. Therefore, descriptive synthesis of the studies was undertaken. The two studies presented mixed results regarding the effect on bodily pain. While Jena et al. (2020) [40] demonstrated a beneficial effect of exercises on mitigating pain, Bosnak-Guclu et al. (2011) [35] indicated a nonsignificant effect.

In addition, one study [31] investigated the effects of exercise on gastrointestinal symptoms and peripheral circulatory symptoms (i.e., a combination of muscle fatigue, edema, cold legs and hands). The study employed a three-arm design (i.e., group A, physical training with constant workload vs. group B, physical training with progressive/increasing workload vs. group C, no-training control group) and subscales from the Subjective Symptoms Assessment Profile were used for evaluation. Results from this study indicated that physical exercise training could improve peripheral circulatory symptoms in adults with HF (*p* < 0.01) and progressive/increasing workload seemed to be superior to constant workload. Yet, the study did not find significant effects on gastrointestinal symptoms (*p* = 0.19).

## 4. Discussion

Our systematic review summarized data from 17 RCTs covering 1086 participants to estimate the effects of exercise-based interventions on fluid overload symptoms in patients with HF. We found robust evidence indicating that exercise-based interventions significantly improved dyspnea compared to no-exercise usual care. This systematic review also found that the intervention length acted as an effect modifier that impacted the treatment effect and assisted in explaining the between-study heterogeneity. More specifically, the treatment effect on dyspnea were gradually diminished as the intervention went longer. Edema also seemed to be alleviated by exercise-based interventions, yet more convincing studies are required to confirm this finding. Notably, the interventions did not improve fatigue compared with usual care and the nonsignificant finding was not influenced by any of the predetermined trial-level characteristics considered in the meta-regression. High-quality research has established exercise as an effective intervention to reduce cancer-related fatigue [48,49,50,51]. Future research is needed to investigate the mechanism of HF-related fatigue and the relationship between fatigue and fluid overload due to HF. The paucity of studies and heterogeneity of symptom measures used in the studies limited the review in determining the effects of exercise-based interventions on other fluid overload symptoms, including bodily pain, gastrointestinal symptoms, and peripheral circulatory symptoms. Overarching conclusions regarding which type of exercise (respiratory muscle contracted exercises vs. peripheral muscle contracted exercises vs. both respiratory and peripheral muscle contracted exercises) is superior in achieving positive outcomes cannot be drawn from our subgroup analyses in this review. This possibly reflects either a true lack of differences between subgroups or a limited number of trials within each subgroup to reach statistical power [26]. Nevertheless, the review findings provide meaningful insights into the components and efficacy of current exercise-based interventions to manage fluid overload symptoms in patients with HF.

Dyspnea, edema, and fatigue are hallmark fluid overload symptoms of HF [52] that have been used to generate a score for grading the degree of congestion in the EVEREST and OPTIMIZE-HF trials [5,6]. The beneficial role of respiratory muscle contracted exercises in alleviating dyspnea in our review is congruent with the results of previous literature reviews [53,54,55]. The whole-body lymph fluid has to be drained through the thoracic or the right lymphatic duct, where lymph eventually returns to the venous circulation via the subclavian vein [16]. Respiratory muscle contracted exercises could alter intrathoracic pressure, stimulate lymphatic ducts, and facilitate lymph fluid drain, thus helping reduce fluid overload, particularly in the thoracic part [56]. Improved tissue volume homeostasis would benefit in mitigating pulmonary congestion and symptomatic relief of dyspnea. Respiratory muscle contracted exercises could, meanwhile, benefit patients by an improvement in corresponding muscle strength and endurance, hence contributing to a better respiratory function and decreased breathlessness [55]. Moreover, our review demonstrated that peripheral muscle contracted exercises appeared to have similar effect in alleviating dyspnea. To our knowledge, this is a comparatively new result. The mechanisms that regulate lymph propulsion might underlie the observed effect. Peripheral muscle contracted exercises such as walking and bicycling induce not only musculoskeletal contractions, but also breathing alterations, arterial pulsations, skin tensions, and postural changes. These biological adaptions help to activate lymphatic systems and promote lymph propulsion throughout the overall body, thereby leading to a reduction of systemic fluid overload in interstitial spaces and amelioration of dyspnea [16,56]. On the other hand, peripheral muscle contracted exercises have been consistently shown to enhance oxygen uptake and ventilatory efficiency without worsening cardiac function in patients with HF [57]. These indicators acting as useful measures of cardiorespiratory fitness and breathing status likewise reflected the promising role of such exercises in dyspnea alleviation. In addition, the results of meta-regression further found that the positive effect of exercises on dyspnea might be gradually weakened as the intervention lasted longer. Indirect support for this finding can be drawn from previous studies [21,58], which have suggested that lymph flow from active skeletal muscle was not maintained constantly during the course of steady-state exercise, instead, after a high initial increase at the beginning the lymph flow velocity was gradually attenuated as the prescribed exercise continued. Efficient lymphatic drainage requires the coordination of lymphatic pumping. Specifically, lymphatic contractions occur with a phase lag as the contractions propagated downstream from one lymphangion to the next produced the highest lymph flow [12,15]. Recent research demonstrated that lymphatic exercises designed to mimic lymphatic pumping to promote lymph flow are effective to reduce lymph fluid level, lymphatic pain, swelling, and reverse the mild lymphedema due to breast cancer [56,59,60]. Future research may test the lymphatic exercises on fluid overload symptoms in patients with HF. Another explanation could be that patient adherence has a fundamental role in the impact of the resulting benefits, but their participation and compliance with the exercise protocols tend to diminish over a long intervention period. In consequence, the exercise effect could be gradually weakened.

Our review suggested that peripheral muscle contracted exercises (aerobic exercises) reduced the fluid overload symptom of edema. All edema develops due to an imbalance between capillary filtration and lymph drainage, and lymph transport is the main process responsible for interstitial lymph fluid drainage [61]. Theoretically, an improvement in lymph drainage is of value in all forms of peripheral edema. Unfortunately, no effective drug therapy exists for accelerating lymph flow at present. Movement and particular exercise, by inducing alternating changes in interstitial fluid pressure, increase initial lymphatic filling and, hence, lymph flow [61]. As a result, the dynamic physiological forces elicit patient edema relief. Despite the underpinning physiological mechanisms, convincing empirical evidence of exercises’ effectiveness for peripheral edema is very limited. Only one trial was included for this symptom outcome in our review [40]. Further well-designed studies are required to reinforce the result.

Notably, exercise-based interventions did not significantly improve fatigue as compared to no-exercise control groups, neither the peripheral muscle contracted exercises nor the respiratory muscle contracted exercises. Yet, the nonsignificant effect in fatigue was not stable as we removed the study by Wall et al. (2010) [44]. From the forest plot, we can see that the direction and magnitude of effect size of Wall et al. (2010) [44] is divergent with the rest of the included studies. In regard to this unanticipated nonsignificant result, Wall et al. (2010) [44] explained that “the de-conditioned individuals in the intervention group must go through a period of reconditioning while exercising, and physical activity can lead to fatigue as with any healthy individual who exercises, until an individual becomes physically fit enough to endure a specific level of training”. This is a plausible argument for a nonsignificant change in perceived fatigue for this kind of patients. On the other side, fatigue reflects muscle hypoperfusion and an indicator of reduced cardiac output [62]. From the perspective of lymphatic dynamics, exercises create muscle milking and pumping to promote overall body lymph fluid flow and drain [56]. A decrease in tissue fluid content by as little as 2.5% can lead to a 30%–40% increase in cardiac output [63]. Increased cardiac output ensures blood perfusion of not only the vital organs (e.g., heart and brain) but also less vital organs (e.g., limbs), thus preventing or decreasing the perception of fatigue from musculoskeletal system [62]. Overall, based on currently available evidence, we cannot yet draw a definitive conclusion about the intervention effect on fatigue. Interestingly, from the forest plot we found that Baduanjin exercise in contrast with other exercise modalities achieved an evident and significant effect in fatigue relief [45]. On this basis, we speculate that peripheral muscle contracted exercises, if coordinated with synchronized breathing exercises (e.g., lymphatic exercises) may elicit health benefits without apparent perception of being fatigue.

In this systematic review, we also included data for other symptoms that are physiologically related to fluid overload, including bodily pain, gastrointestinal symptoms, as well as peripheral circulatory symptoms, but the limited available data precluded quantitative synthesis that allowed any firm conclusions to be made. Nevertheless, this initial work paves the path for future studies to gain deep insights into the effects of exercise-based interventions for a spectrum of fluid overload symptoms.

### Limitations of Existing Literature and Strengths of The Study

Several limitations were identified through this systematic review in existing literature. First, the overall risk of bias for the included studies was predominately of “some concerns”. Details of random sequence generation and allocation concealment and blinding of outcome assessments were particularly poorly described, as such, the involved studies were likely subjected to selection or detection bias. Second, apparent inadequacies in the description of participant characteristics and intervention process impeded us from gathering sufficient data to ascertain the potential effect modifiers. For example, previous studies have identified BMI as a significant predictor of lymphatic function [64,65], and accordingly we considered it as one of the predetermined factors that could possibly modify the observed treatment effects. However, only three included studies provided participants’ BMI value, which did not meet the requirement of a minimum number of studies to proceed with the meta-regression analysis. Third, there are no well-established instruments to measure fluid overload symptoms in HF, as a consequence, the included studies varied considerably in assessment tools used, which would undoubtedly add inconsistency to our estimates. So, caution is warranted in interpreting the results of this systematic review.

This is the first review of available literature, to our knowledge, that attempts to provide insights into the effects of exercise-based interventions on fluid overload symptoms in patients with HF. Fluid overload symptoms are highly prevalent and burdensome, profoundly limiting patients’ daily functioning and well-being [4], but no previous review has focused specifically on these patient-centered outcomes. A major strength of the review lies in its novelty of looking at the role of therapeutic exercises in HF symptom management from a perspective of lymphatic system. The central role of the lymphatic system in tissue fluid balance has been historically ignored, mainly due to the challenge of visualizing the transparent lymphatic vessels [13]. Recognition of activating lymphatic pumping to counteract fluid accumulation and symptom onset is significant as the removal of interstitial fluid from tissues is performed exclusively by the lymphatic system [16] and the system is highly adaptable and sensitive to small alterations in internal or external pressure [17]. Another strength of the review is the application of the most rigorous methods appropriate for the research question of interest. We adhered to standard Cochrane methodological procedures to minimize potential biases throughout the systematic review process [26]. Only RCTs were included to ensure the quality of the body of evidence. Sensitivity analysis was performed to evaluate the stability of results. Furthermore, we applied heterogeneity tests to explore differences across the prespecified subgroups of various types of exercise and meta-regressions to explore whether the treatment effects could depend on certain participant characteristics, intervention characteristics, or the trial characteristic.

## 5. Conclusions

Symptom escalation is one of the most distressing scenarios that patients encounter along their HF trajectories and the most commonly cited reason for hospital readmission [66]. Efficacious symptom control is a major focus in HF. Our synthesized evidence generally supports the promising role of exercise-based interventions as a non-pharmaceutical therapy for management of dyspnea and edema. Meanwhile, intervention length can act as an effect modifier in the treatment effects of exercise training that maintains a constant intensity and frequency. While evidence was not conclusive for fatigue, our study provided a possible explanation that peripheral muscle contracted exercises coordinated with controlled breathing exercises may elicit health benefits with apparent alleviation of fatigue during the exercises. Future studies are warranted with high methodological quality and comprehensive assessment of fluid overload symptoms as well as objective measure of fluid overload. The evidence gap concerning other fluid overload symptoms also needs further investigation.

## Figures and Tables

**Table 2:** Univariate meta-regression analysis.

	Dyspnea			Fatigue		
	No.	Regression Coefficient (95% CI)	*p* Value	No.	Regression Coefficient (95% CI)	*p* Value
Mean age (years)	9	0.006 (−0.065, 0.076)	0.85	6	−0.006 (−0.143, 0.131)	0.91
Male percentage (%)	9	−0.001 (−0.033, 0.032)	0.97	6	0.006 (−0.051, 0.064)	0.78
BMI (kg/m^2^)	3	-	-	3	-	-
LVEF (%)	6	0.050 (−0.121, 0.221)	0.46	4	-	-
NYHA	8	−0.003 (−0.020, 0.013)	0.62	5	-	-
Intervention length (weeks)	9	0.033 (0.003, 0.063)	0.04	6	0.007 (−0.019, 0.034)	0.47
Intervention dose ^a^	9	−0.004 (−0.033, 0.026)	0.79	6	0.007 (−0.031, 0.046)	0.63
Supervised ^b^	9	0.091 (−0.846, 1.028)	0.83	6	0.564 (−0.839, 1.967)	0.33
Setting						
Home-based	4	0.176 (−0.800, 1.152)	0.68	2	0.200 (−2.140, 2.540)	0.80
Mixed settings ^c^	2	−0.292 (−1.378, 0.794)	0.54	2	−0.222 (−2.485, 2.042)	0.78
Center-based ^d^	3	Ref.	-	2	Ref.	-
Study quality						
Low risk of bias	2	−0.307 (−1.744, 1.129)	0.62	2	Ref.	-
Some concerns	6	0.105 (−1.203, 1.414)	0.85	4	0.276 (−1.161, 1.714)	0.62
High risk of bias	1	Ref.	-	0	-	-

Note: ^a^ Intervention dose calculated by intervention length times no. sessions per week times session length. ^b^ Supervised categorized according to the exercise sessions under constant supervision or partial supervision versus those not under any supervision. ^c^ Mixed settings defined as center-based setting in combination with some home exercise sessions. ^d^ Center-based settings defined as exclusively hospital-based, rehabilitation center-based or health care exercise facility-based exercise sessions. Abbreviations: CI, confidence interval; BMI, body mass index; LVEF, left ventricular ejection fraction; NYHA, New York Heart Association.

## Data Availability

The data presented in this study are available on request from the corresponding author.

## Supplementary Materials

The following supporting information can be downloaded at: https://www.mdpi.com/article/10.3390/biomedicines10051111/s1, Supplementary Chart S1: Search strategy; Supplementary Table S1: Characteristics of included studies; Supplementary Figure S1: Preliminary meta-analysis for dyspnea; Supplementary Figure S2: Preliminary meta-analysis for fatigue.
