# Supplementary material for: The Effects of Exercise-Based Interventions on Fluid Overload Symptoms in Patients with Heart Failure: A Systematic Review and Meta-Analysis
Source: Biomedicines. 2022 May 11;10(5):1111. doi: 10.3390/biomedicines10051111 (PMC9138396; doi:10.3390/biomedicines10051111)

Supplementary Chart S1: Search Strategy

- **MEDLINE Ovid**

1. exp heart failure/
2. ((cardi* or heart* or myocard*) adj2 (fail* or incompet* or insufficien* or decomp* or weak* or dysfunction)).ti,ab.
3. **1 or 2**
4. exp Exercise Therapy/
5. exp Exercise/
6. *Rehabilitation/
7. rehabilitat$5.ti,ab.
8. (physical$4 adj3 (fit or fitness or train$5 or therap$5 or activit$5)).ti,ab.
9. ((train$5 or exercise$4) adj3 (strength$3 or resistance or endurance or muscle or inspiratory or aerobic or breath$3 or interval)).ti,ab.
10. ((exercise$4 or fitness) adj3 (treatment or intervent$4 or program$2 or therapy)).ti,ab.
11. ((lifestyle or life-style) adj3 (intervent$5 or program$2 or treatment$2)).ti,ab.
12. **or/4-11**
13. exp DYSPNEA/
14. (dyspn?e* or orthopn?ea or breath*).ti,ab.
15. Respiratory Sounds/
16. exp COUGH/
17. (cough* or wheez*).ti,ab.
18. exp Edema, Cardiac/
19. (edema* or oedema*).tw.
20. (swelling or swollen or effus* or bloat* or congest* or fatigue or bendopnea or rale*).ti,ab.
21. ((fluid or volume or water or hydric or hydration) adj2 (retention or retain* or accumulat* or congest* or overload* or buil* or redistribut*)).ti,ab.
22. (Weight* or Body Mass Index or BMI or heaviness).ti,ab.
23. **or/13-22**
24. randomized controlled trial.pt.

25. controlled clinical trial.pt.
26. randomized.ab.
27. placebo.ab.
28. drug therapy.fs.
29. randomly.ab.
30. trial.ab.
31. groups.ab.
32. **or/24-31**
33. exp animals/ not humans.sh.
34. **32 not 33**
35. **3 and 12 and 23 and 34**

- **Embase Ovid**

1. exp heart failure/
2. ((cardi* or heart* or myocard*) adj2 (fail* or incompet* or insufficien* or decomp* or weak* or dysfunction)).ti,ab.
3. **1 or 2**
4. exp Exercise Therapy/
5. exp Exercise/
6. *Rehabilitation/
7. rehabilitat$5.ti,ab.
8. (physical$4 adj3 (fit or fitness or train$5 or therap$5 or activit$5)).ti,ab.
9. ((train$5 or exercise$4) adj3 (strength$3 or resistance or endurance or muscle or inspiratory or aerobic or breath$3 or interval)).ti,ab.
10. ((exercise$4 or fitness) adj3 (treatment or intervent$4 or program$2 or therapy)).ti,ab.
11. ((lifestyle or life-style) adj3 (intervent$5 or program$2 or treatment$2)).ti,ab.
12. **or/4-11**
13. exp DYSPNEA/
14. (dyspn?e* or orthopn?ea or breath*).ti,ab.

15. Respiratory Sounds/
16. exp COUGH/
17. (cough* or wheez*).ti,ab.
18. exp Edema, Cardiac/
19. (edema* or oedema*).ti,ab.
20. (swelling or swollen or effus* or bloat* or congest* or fatigue or bendopnea or rale*).ti,ab.
21. ((fluid or volume or water or hydric or hydration) adj2 (retention or retain* or accumulat* or congest* or overload* or buil* or redistribut*)).ti,ab.
22. (Weight* or Body Mass Index or BMI or heaviness).ti,ab.
23. **or/13-22**
24. (random* or factorial* or crossover* or cross over* or cross-over* or placebo* or (doubl* adj blind*) or (singl* adj blind*) or assign* or allocat* or volunteer*).tw.
25. crossover procedure/ or double blind procedure/ or randomized controlled trial/ or single blind procedure/
26. **24 or 25**
27. (animal/ or nonhuman/) not human/
28. **26 not 27**
29. **3 and 12 and 23 and 28**

- **CENTRAL**

#1 MeSH descriptor: [Heart Failure] explode all trees

#2 ((cardi* or heart* or myocard*) near/2 (fail* or incompet* or insufficien* or decomp* or weak* or dysfunction))

#3 #1 or #2

#4 MeSH descriptor: [Exercise Therapy] explode all trees

#5 MeSH descriptor: [Exercise] explode all trees

#6 MeSH descriptor: [Rehabilitation] this term only

#7 rehabilitat*

#8 physical* near/3 (fit or fitness or train* or therap* or activit*)

#9 (train* or exercis*) near/3 (strength* or resistance or endurance or muscle or inspiratory or aerobic or breath* or interval)

#10 (exercis* or fitness) near/3 (treatment or intervent* or program* or therapy)

#11 (lifestyle or life-style) near/3 (intervent* or program* or treatment*)

#12 #4 or #5 or #6 or #7 or #8 or #9 or #10 or #11

#13 MeSH descriptor: [Dyspnea] explode all trees

#14 dyspn?e* or orthopn?ea or breath*

#15 MeSH descriptor: [Respiratory Sounds] explode all trees

#16 MeSH descriptor: [Cough] explode all trees

#17 cough* or wheez*

#18 MeSH descriptor: [Edema, Cardiac] explode all trees

#19 edema* or oedema*

#20 swelling or swollen or effus* or bloat* or congest* or fatigue or bendopnea or rale*

#21 (fluid or volume or water or hydric or hydration) near/2 (retention or retain* or accumulat* or congest* or overload* or buil* or redistribut*)

#22 Weight* or Body Mass Index or BMI or heaviness

#23 #13 or #14 or #15 or #16 or #17 or #18 or #19 or #20 or #21 or #22

#24 #3 and #12 and #23

- **CINAHL EBSCO**

S1 MH "heart failure+"

S2 TI ((cardi* or heart* or myocard*) N2 (fail* or incompet* or insufficien* or decomp* or weak* or dysfunction))

S3 S1 or S2

S4 MH "Therapeutic Exercise+"

S5 MH "Exercise+"

S6 MH "Rehabilitation"

S7 rehabilitat*

S8 physical* N3 (fit or fitness or train* or therap* or activit*)

S9 (train* or exercise*) N3 (strength* or resistance or endurance or muscle or inspiratory or aerobic or breath* or interval)

S10 (exercise* or fitness) N3 (treatment or intervent* or programs* or therapy)

S11 (lifestyle or life style) N3 (intervent* or program* or treatment*)

S12 S4 OR S5 OR S6 OR S7 OR S8 OR S9 OR S10 OR S11

S13 MH "DYSPNEA+"

S14 dyspnea or dyspnoea or orthopnea or orthopnoea or breath*

S15 MH "Respiratory Sounds"

S16 MH "Cough+"

S17 cough* or wheez*

S18 MH "edema+"

S19 edema* or oedema*

S20 swelling or swollen or effus* or bloat* or congest* or fatigue or bendopnea or rale*

S21 (fluid or volume or water or hydric or hydration) N2 (retention or retain* or accumulat* or congest* or overload* or buil* or redistribut*)

S22 Weight* or Body Mass Index or BMI or heaviness

S23 S13 OR S14 OR S15 OR S16 OR S17 OR S18 OR S19 OR S20 OR S21 OR S22

S24 (MH "Clinical Trials+") OR (PT clinical trial OR TX ((clinic* N1 trial?) OR (singl* N1 blind*) OR (doubl* N1 blind*) OR (singl* N1 mask*) OR (doubl* N1 mask*) OR random*) OR (MH "Placebos") OR TX (placebo* OR assign* OR control* OR allocat*) OR (MH "Crossover Design") OR TX (volunteer* OR crossover* OR cross-over*))

S25 S3 AND S12 AND S23 AND S24

Supplementary Table S1: Characteristics of Included Studies

Author, Year (Country)	Setting and Sample Size	Participants †	Interventions		Outcomes (Measures) /Assessment Timepoints
			Exercise-based Interventions	Comparison	
Alkan, et al., 2017 (Turkey) [1]	Single center (Cardiology Institute of Istanbul University)<br><br>N randomized total: N=70 35 (I) vs. 35 (C) N analyzed total: N=56; 29 (I) vs. 27 (C)<br><br>Attrition rate: 20.0% (I: 6/35; C: 8/35)	Mean age (SD), years: 64.9 (13.1) vs. 63. 9 (12.7) total: 64.4 (12.8)<br>Female, n (%): 15 (51.7%) vs. 19 (70.4%) total: 34 (60.7%)<br>White, n (%): NR<br>Mean BMI (SD), kg/m^2: NR<br>HF etiology, n (%): NR<br>NYHA class, n (%): II: 15 (26.8%) III: 41 (73.2%)<br>Mean LVEF (SD), %: NR	Intervention: Breathing exercise<br>Content: (1) Breathing exercises (diaphragmatic and pursed lip respirations) were taught to patients by demonstration and explanation technique. Pursed lip respiration is to make expiration in a slow way with pursed lips. Diaphragmatic respiration technique could increase efficiency of diaphragm as an inspiratory muscle and thus help make respiration deeper and more effective. Diaphragmatic respiration technique was started to be applied after pursed lip respiration technique, that's to say when patient experienced less dyspnea and had more control on respiration. (2) A written text describing breathing exercises was given to patients. (3) Three times of phone interview was made and support was given to patients in the experiment group.<br>**[Respiratory muscle contracted]**<br>Frequency: 30 minutes daily.<br>Duration: 3 months.<br>Intensity: NR<br>Setting: Home.<br>Deliverer: NR<br>Involvement of family: NR<br>Other: N/A	The control group received no education or demonstration on breathing techniques.	(1) Dyspnea (Basal Dyspnea Index, BDI) /baseline & 3 months
Beniaminovitz, et al., 2002 (United States) [2]	Single center (Columbia Presbyterian Hospital)	Mean age (SD), years: 50 (3) vs. 48 (4) total: 49.36 (3.4)<br>Female, n (%):	Intervention category: Low-level leg muscle training<br>Content: (1) Supervised training sessions were conducted 3 times/week and consisted of 15 min each of bicycle and treadmill exercise at a workload corresponding to 50% of peak VO_2 with a minute ventilation <25 l/min and a	Patients in active control group learned guided imagery relaxation techniques.	(1) Dyspnea (the Transitional Dyspnea Scale, TDI) /3 months

	N randomized<br>total: N=29<br>20 (I) vs. 9 (C)<br>N analyzed<br>total: N=25;<br>17 (I) vs. 8 (C)<br><br>Attrition rate:<br>13.8% (I: 3/20; C: 1/9)	5 (29.4%) vs. 2 (25%)<br>total: 7 (28%)<br>White, n (%):<br>NR<br>Mean BMI (SD), kg/m^2:<br>NR<br>HF etiology, n (%):<br>Ischemic: 13 (52%)<br>Dilated: 12 (48%)<br>Mean NYHA class (SD):<br>NR<br>Mean LVEF (SD), %:<br>20 (1) vs. 18 (1)<br>total: 19.36 (1.37)	heart rate (HR) <120 beats/min. (2) A series of leg calisthenics were performed, including hip flexion and extension with ankle weights (5 lb/leg) and thigh muscle contraction using therabands. Each month, an additional set of exercises was added and ankle weights were increased by 2 lb/leg. The resistance of the theraband was also increased.<br>**[Peripheral muscle contracted]**<br>Frequency: 3 times per week.<br>Duration: 3 months.<br>Intensity: Workload corresponding to 50% of peak VO_2 with a minute ventilation < 25 l/min and a heart rate (HR) <120 beats/min.<br>Setting: NR<br>Deliverer: NR<br>Involvement of family: NR<br>Other: The selective exercise of leg muscles at a low level did not condition the respiratory muscles.	The patients attended 90-min, weekly therapist-guided sessions for 1 month and were taught a series of relaxation/imagery exercises that promoted stress reduction. Patients were encouraged to perform the mental exercises twice daily.	
Bosnak-Guclu, et al., 2011 (Turkey) [3]	Single center (Hacettepe University Faculty of Health Sciences, Department of Physiotherapy and Rehabilitation)<br><br>N randomized<br>total: N=36<br>18 (I) vs. 18 (C)<br>N analyzed<br>total: N=30<br>16 (I) vs. 14 (C)	Mean age (SD), years:<br>69.5 (8.0) vs. 65.7 (10.5)<br>total: 67.7 (9.3)<br>Female, n (%):<br>4 (25.0%) vs. 2 (14.3%)<br>total: 6 (20%)<br>White, n (%):<br>NR<br>Mean BMI (SD), kg/m^2:<br>26.8 (4.3) vs. 25.1 (3.2)<br>total: 25.9 (3.8)<br>HF etiology, n (%):<br>Ischemic: 26 (86.7%)<br>Nonischemic: 4 (13.3%)<br>NYHA class, n (%):	Intervention category: Inspiratory muscle training<br>Content: (1) All patients were taken in one-week familiarization period and instructed to learn breathing adequately with a threshold inspiratory muscle training device. (2) The treatment group received inspiratory muscle training at 40% of the maximal inspiratory pressure. Maximal inspiratory pressure was measured at a supervised session each week, and 40% of measured value was the weekly new training workload. (3) Once a day at each week, patients' heart rate, blood pressure and breathing frequency were monitorized during the training sessions and new workload was adjusted in the treatment group. (4) Patients were instructed to maintain diaphragmatic breathing, and try to maintain 10-15 breaths and rested 5-10s between breaths. As soon as the patients managed; they were encouraged to maintain 25-30 breaths at each workload. (5) Patients were checked by phone calls tiwce a week, whether they are doing inspiratory muscle training in a right way. (6) All patients wore nose-clip during sessions. Training diaries were checked weekly.	The sham control group received (1) and (5), and inspiratory muscle training at a fixed workload of 15% of the maximal inspiratory pressure.	(1) Dyspnea (Modified Medical Research Council Dyspnea Scale, MMRC)<br>/baseline & 1.5 months<br>(2) Fatigue (Turkish version of the Fatigue Severity Scale, Turkish FSS)<br>/baseline & 1.5 months<br>(3) Pain (SF-36 bodily pain dimension)<br>/baseline & 1.5 months

	Attrition rate: 16.67% (I:2/18; C: 4/18)	II: 20 (66.7%)<br>III: 10 (33.3%)<br>Mean LVEF (SD), %:<br>33.4 (7.2) vs. 36.1 (7.6)<br>Total: 34.9 (7.4)	**[Respiratory muscle contracted]**<br>Frequency: 30 min-per day, 7 days per week, for 6 weeks.<br>Duration: 1.5 months.<br>Intensity: 40% of the maximal inspiratory pressure.<br>Setting: Six sessions were performed at home and 1 session was supervised at the rehabilitation department in both groups at each week.<br>Deliverer: Physiotherapists.<br>Involvement of family: NR<br>Other: The subjects were told not to exercise or do physical activity over their normal routine during the study period.		
Chen, et al., 2018 (Taiwan) [4]	Two centers (Two large medical centers in northern Taiwan)<br><br>N randomized<br>total: N=80<br>39 (I) vs. 41 (C)<br>N analyzed<br>total: N=63<br>30 (I) vs. 33 (C)<br><br>Attrition rate: 21.25% (I: 9/39; C: 8/41)	Mean age (SD), years:<br>69.1 (13.5) vs 71.4 (13.7)<br>total: 70.3 (13.5)<br>Female, n (%):<br>21 (53.8%) vs. 17 (41.5%)<br>total: 38 (47.5%)<br>White, n (%):<br>NR<br>Mean BMI (SD), kg/m^2:<br>NR<br>HF etiology, n (%):<br>NR<br>NYHA class, n (%):<br>I: 21 (26.2%)<br>II: 59 (73.8%)<br>Mean LVEF (SD), %:<br>56.7 (17. 6) vs. 60. 4 (13.4)<br>Total: 58.6 (15.6)	Intervention category: Baduanjin exercise program<br>Content: (1) The treatment group underwent a Baduanjin exercise program. The program included a 35-minute demonstration video, a picture-based educational brochure and a performance record form. (2) The Baduanjin exercise included three phases: warm-up, exercise, and cool-down. During the warm-up and cool-down phases, the participants performed deep breathing and stretching exercises to improve range of motion in their joints. During the exercise phase, the participants performed eight sections of movements led by the Baduanjin exercise video. (3) Participants were asked to record their daily exercise schedule on a performance record form for 12 weeks. Weekly telephone follow-up was performed by the researcher to monitor the participants' exercise performance.<br>**[Both respiratory & peripheral muscle contracted]**<br>Frequency: 35-minute per time, three times per week for 12 weeks.<br>Duration: 3 months.<br>Intensity: NR<br>Setting: Home.<br>Deliverer: The researcher, a trained Baduanjin instructor.<br>Involvement of family: NR<br>Other: The 35-minute Baduanjin exercise demonstration video was	The control group received usual care and underwent no intervention. Participants in the control group were advised to maintain their usual lifestyle and completed no exercise programs.	(1) Fatigue (Chinese version of the shorted Piper Fatigue Scale, Chinese PFS)<br>/baseline, 1, 2 & 3 months

			produced by the Chinese Traditional Exercise Association for use by patients with HF in this study. Baduanjin can promote the functions of multiple organs and system through regulation of breathing and movement of different joints and muscles.		
Corvera-Tindel, et al., 2004 (United States) [5]	Two centers (Veterans Affairs medical center and a university-affiliated medical center)<br><br>N randomized total: *N*=79<br>42 (I) vs. 37 (C)<br>N analyzed total: N=63<br>32 (I) vs. 31 (C)<br><br>Attrition rate: 20.25% (I: 10/42; C: 6/37)	Mean age (SD), years:<br>63.8 (10.1) vs. 61.3 (11.1)<br>total: 62.6 (10.6)<br>Female, n (%):<br>0 (0%) vs. 1 (3%)<br>total: 1 (1.3%)<br>White, n (%):<br>24 (57%) vs. 13 (35%)<br>total: 37 (46.8)<br>Mean BMI (SD), kg/m^2:<br>30.1 (6.4) vs. 28.8 (6.1)<br>total: 29.5 (6.3)<br>HF etiology, n (%):<br>Ischemic: 39 (49.4%)<br>Nonischemic: 40 (50.6%)<br>NYHA class, n (%):<br>II: 63 (79.7%)<br>III/IV: 15 (19.0%)<br>Mean LVEF (SD), %:<br>29.1 (8.5) vs. 24.7 (8.8)<br>Total: 27 (8.8)	Intervention category: Home walking exercise<br>Content: (1) The treatment group underwent home walking exercise initiated at 10 min and 40% of maximal heart rate and progressively increased up to 60 minutes and 65% maximal heart rate in the last 6 weeks of the program. (2) During each weekly home visit, the nurse 1) provided a weekly walking prescription including duration, frequency, and intensity, 2) walked with the patient to evaluate exercise tolerance and validate self-reported protocol compliance, 3) conducted a brief physical assessment immediately before and after walking exercise, and 4) reviewed pedometer data and counseled patient about incomplete data.<br>**[Peripheral muscle contracted]**<br>Frequency: Once a day, 5 days per week; each session was initiated at 10 min and progressively increased to 60 min.<br>Duration: 3 months.<br>Intensity: Initiated at 40% of maximum heart rate and progressively increased up to 60 min and 65% maximal heart rate in the last 6 weeks of the program.<br>Setting: Home.<br>Deliverer: A Nurse.<br>Involvement of family: NR<br>Other: Patients in both groups received a pedometer, a small device anchored to a patient's lower leg and calibrated to his/her stride to record time and distance walked (i.e., number of steps and miles).	The control group patients were instructed to maintain their normal daily activities and asked not to begin a regular exercise program. During each home visit, the nurse 1) obtained vital signs and performed a brief physical assessment, 2) reinforced maintenance of daily activities, and 3) reviewed all-day pedometer data.	(1) Dyspnea (Dyspnea-Fatigue Index, DFI)<br>/3 months

Hossein Pour, et al., 2020 (Iran) [6]	Two centers (two cardiac rehabilitation hospitals in two teaching hospitals affiliated to the Lorestan University of Medical Sciences, in the west of Iran)<br><br>N randomized total: *N*=98<br>49 (I) vs. 49 (C)<br>N analyzed total: N=84<br>42 (I) vs. 42 (C)<br><br>Attrition rate: 14.29% (I: 7/49 C: 7/49)	Mean age (SD), years:<br>55.97 (9.43) vs. 57.28 (9.06), total: 56.63 (9.21)<br>Female, n (%):<br>19 (45.2%) vs. 21 (50%), total: 40 (47.6%)<br>White, n (%):<br>NR<br>Mean BMI (SD), kg/m^2:<br>25.6 (5.2) vs. 25.8 (4.1), total: 25.7 (4.7)<br>HF etiology, n (%):<br>Ischemic: 24 (28.6%)<br>Dilated: 60 (61.4%)<br>NYHA class, n (%):<br>II: 32 (38.1%)<br>III: 42 (50.0%)<br>IV: 10 (11.9%)<br>Mean LVEF (SD), %:<br>33.7 (6.1) vs. 32.5 (4.4), Total: 33.1 (5.3)	Intervention category: Inspiratory muscle training<br>Content: (1) The treatment group received inspiratory muscle training at 40% of the maximal inspiratory pressure and its training loads were adjusted to maintain 40% of the maximal inspiratory pressure weekly. (2) The treating nurses measured the values of maximal inspiratory pressure every week during the treatment and readjusted the training workloads. (3) The patients were encouraged to maintain diaphragmatic breathing for 10~15 breaths and to take 5~10 s of rest between their breaths. (4) The patients were followed up on the phone twice a week to check the correct performance of the exercises and potential problems or adverse effects during training.<br>**[Respiratory muscle contracted]**<br>Frequency: 30 min per day (once a day), 7 days per week, for 6 weeks. Every 30-min session per day included 3-min sets of training, followed by short intervals of 1-min rest between the sets.<br>Duration: 1.5 months.<br>Intensity: 40% of the maximal inspiratory pressure.<br>Setting: Rehabilitation center and home. For every week, six sessions of the training were performed at home in both groups, and one supervised session was at the rehabilitation centers (one cardiac nurse in each center).<br>Deliverer: Two cardiac nurses.<br>Involvement of family: NR<br>Other: N/A	The sham control group received inspiratory muscle training at a fixed workload of 10% of the maximal inspiratory pressure.	(1) Dyspnea (the Modified Medical Research Council, MMRC) /baseline & 1.5 months<br>(2) Fatigue (the Fatigue Severity Scale, FSS) /baseline & 1.5 months
Jena, et al., 2020 (India) [7]	Single center (Cardiology ward of IMS & SUM Hospital, Bhubaneswar, Odisha)<br><br>N randomized	Age range, years, %:<br>30-39: 12.5%<br>40-49: 17.5%<br>50-59: 25%<br>>60: 45%<br>Female, n (%):<br>15 (37.5%)<br>White, n (%):	Intervention category: Aerobic exercise<br>Content: Other than conservative treatment, the training group was trained with 30 minutes of aerobic exercises for 30 days. If the patients were discharged from the hospital before 30 days of training, they were followed up through telephonic conversation and the home visit in the evening. In the study, aerobic exercise was defined as stretching exercise, walking, and bicycling from low to high intensity.<br>**[Peripheral muscle contracted]**	The control group received conservative treatment only.	(1) Edema of dependent extremities (Edema grading scale) /1 month<br>(2) Chest pain (Numeric pain rating scale) /1 month

	total: *N*=40<br>20 (I) vs. 20 (C)<br>N analyzed<br>total: *N*=40<br>20 (I) vs. 20 (C)<br><br>Attrition rate:<br>NR	NR<br>Mean BMI (SD), kg/m^2:<br>NR<br>HF etiology, n (%):<br>NR<br>Mean NYHA class (SD):<br>NR<br>Mean LVEF (SD), %:<br>NR	Frequency: 30 minutes, daily.<br>Duration:1 month.<br>Intensity: NR<br>Setting: Hospital or in the patient's home through telephonic conversation and home visits.<br>Deliverer: NR<br>Involvement of family: NR<br>Other: N/A		
Jin & Lee, 2016 (Korea) [8]	Single center<br>(A university hospital in Kyeong-nam area)<br><br>N randomized<br>total: *N*=60<br>32 (I) vs. 28 (C)<br>N analyzed<br>total: *N*=41<br>16 (I) vs. 25 (C)<br><br>Attrition rate:<br>31.67% (I: 16/32; C: 3/28)	Mean age (SD), years:<br>60.1 (7.0) vs. 56.7 (14.4)<br>total: 58.0 (12.0)<br>Female, n (%):<br>3 (18.8%) vs. 8 (32.0%),<br>total: 11 (26.8%)<br>White, n (%):<br>NR<br>Mean BMI (SD), kg/m^2:<br>NR<br>HF etiology, n (%):<br>NR<br>NYHA class, n (%):<br>II: 36 (87.8%)<br>III: 5 (12.2%)<br>Mean LVEF (SD), %:<br>30.0 (6.9) vs. 31.9 (7.0)<br>total: 31.2 (6.9)	Intervention category: Reinforced walking exercise<br>Content: (1) The experimental group was provided with a pedometer and an exercise log, and enhanced walking exercise intervention was performed for 12 weeks. (2) The subjects were encouraged to set weekly goals, perform walking exercise using the pedometer, starting from 5,000 steps in the first week, increased by 10% every week, and the step count was maintained at 10,000 steps or more from the 6th week to the 12th week. (3) Feedback was provided by the researcher, the number of steps/week and the number of steps were checked, the reasons for failure were discussed, and the side effects or adverse reactions that may occur during the 12 weeks were monitorized. (4) Telephone counseling was conducted once a week from week 1 to week 6, and from week 7 to week 12, a total of 9 times were provided once every two weeks. The average time required for a telephone consultation was about 5 to 10 minutes. Text message twice a week (Monday, Thursday).<br>**[Peripheral muscle contracted]**<br>Frequency: 5 days/week.<br>Duration: 3 months.<br>Intensity: The exercise intensity on the Borg scale should be maintained at 12~13 points (slightly difficult), but the appropriate maximum heart rate is about 60~70%.<br>Setting: Home.	The control group maintained hospital treatment by the attending physician (regular medication, regular outpatient visits, and encouraged to practice a healthy lifestyle), and data were collected at the time of data collection in the experimental group.	(1) Dyspnea (Dyspnea-Fatigue Index, DFI)<br>/baseline & 3 months

			Deliverer: A researcher.<br>Involvement of family: NR<br>Other: N/A		
Klocek, et al., 2005 (Poland) [9]	Single center (Cardiac Department of the Medical School of Jagiellonian University)<br><br>N randomized<br>total: N=42<br>14 (I_A) vs. 14 (I_B) vs. 14 (C)<br>N analyzed<br>total: N=42<br>14 (I_A) vs. 14 (I_B) vs. 14 (C)<br><br>Attrition rate: NR	Mean age (SD), years:<br>54 (7) vs. 57 (8) vs. 55 (9), total: 55.9 (8.1)<br>Female, n (%):<br>total: 0 (0%)<br>White, n (%):<br>NR<br>Mean BMI (SD), kg/m^2:<br>27 (2) vs. 28 (4) vs. 26 (5), total: 27 (3.9)<br>HF etiology, n (%):<br>Ischemic: 42 (100%)<br>NYHA class, n (%):<br>II: 18 (42.9%)<br>III: 24 (57.1%)<br>Mean LVEF (SD), %:<br>33.6 (3.6) vs. 34.2 (4.2) vs. 33.2 (3.8)<br>total: 33.7 (3.8)	Intervention category: Physical exercise training<br>Content: Physical training was preceded by a 20-min warming up period, which consisted of general exercises that considered individual musculoskeletal needs.<br>Group I_A—with constant level of workload<br>After warm up, group I_A patients performed a program of training consisting of 4 min cycle ergometer constant workload and 1 min rest, and repeated this cycle 5 times up to 25 min overall training.<br>Group I_B—with progressive/increasing level of workload<br>6 months training period was divided into 3 parts (2 months for each). First phase: After warm up, group I_B patients started with unloaded pedalling with RPM-60 kpm/min for 5 min and then the workload was increased by 25 W every 5 min and lasted 25 min. Second and third phase: The workload was increased (by 10 W) and was limited either by the patient's fatigue or exercise tolerability and heart rate (i.e., 75% of maximal HR for age).<br>Immediately after training, patients from both groups rested listening to relaxing music for the next 15 min in a separate room.<br>**[Peripheral muscle contracted]**<br>Frequency: 25 min per day, 3 days per week.<br>Duration: 6 months.<br>Intensity: 60% of the maximal HR for age in group I_A and 75% of the maximal HR for age in group I_B.<br>Setting: Cardiac Rehabilitation Out-Patient Unit under constant supervision.<br>Deliverer: Physician and rehabilitation specialist.<br>Involvement of family: NR<br>Other: N/A	The controls were not trained and asked not to change their degree of physical activity during the study.	(1) Subjective symptoms including Peripheral circulatory symptoms, G-I symptoms, and Dizziness (the Subjective Symptoms Assessment Profile, SSA-P)<br>/baseline & 6 months

Norman, et al., 2020 (United States) [10]	Two centers (Bryan-LGH Hospital in Lincoln, NE, USA and Henry Ford Hospital in Detroit, MI, USA)<br><br>N randomized total: *N*=204 102 (I) vs. 102 (C)<br>N analyzed total: *N*=159 71 (I) vs. 88 (C)<br><br>Attrition rate: 22.06% (I: 31/102; C: 14/102)	Mean age (SD), years:<br>59.8 (12.6) vs. 60.9 (10.3)<br>total: 60.4 (11.5)<br>Female, n (%):<br>45 (44.1%) vs. 46 (45.1%), total: 91 (44.6%)<br>White, n (%):<br>51 (50.0%) vs 58 (56.9%)<br>total: 109 (53.4%)<br>Mean BMI (SD), kg/m^2:<br>35.0 (8.6) vs. 34.7 (7.8)<br>total: 34.9 (8.2)<br>HF etiology, n (%):<br>NR<br>NYHA class, n (%):<br>I: 16 (7.8%)<br>II: 113 (55.4%)<br>III: 73 (35.8%)<br>IV: 2 (1.0%)<br>Mean LVEF (SD), %:<br>39.3 (12.1) vs. 40.5 (14.0)<br>total: 39.9 (13.1)	Intervention category: HF exercise and resistance training<br>Content: (1) All subjects receive a cardiopulmonary exercise test and 9 supervised exercise training sessions during a 3-week run-in period prior to randomization. (2) The HF Exercise and Resistance Training Camp Intervention (HEART Camp) were provided with free membership to an exercise facility (access) and its general staff (attention) throughout the 18 months of the study. The facility staff were trained to provide customer service (attention) to assist subjects in adjusting exercise equipment to fit the subject and provide safety advice, but did not provide one-on-one exercise counseling or coaching. (3) Subjects were expected to document all moderate intensity exercise in a weekly diary, and wear a heart rate monitor during exercise. (4) The intervention group received ongoing individualized instruction and goal setting with a trained exercise professional who served as a personal coach. (5) The intervention incorporated multicomponent behavioral intervention strategies and consisted of three phases: i. baseline to 6 months (adoption), included developing a relationship with an exercise coach, acclimation and guidance in developing an exercise regime, and participation in weekly meetings with the exercise coach; ii. months 7 to 12 (transitional), included weekly meetings with the exercise coach for ongoing goal-setting and relapse management; iii, months 13 to 18 (maintenance), included weekly reviews of exercise logs by the coach, and only if necessary, a phone call by the coach if a concern was noted or relapse occurred.<br>**[Peripheral muscle contracted]**<br>Frequency: During the maintenance phase, participants are expected to be self-managing exercise and maintaining adherence to 150 minutes of moderate intensity exercise each week. Exercise diaries are submitted weekly, and the coach calls a subject to schedule a face-to-face interaction if relapse occurs during this phase.<br>Duration: 18 months.<br>Intensity: Moderate-intensity aerobic exercise (40%-80% heart rate reserve	Enhanced usual care group received (1), (2) and (3).	(1) Fatigue (Patient-reported outcomes measurement information system, PROMIS-29) /baseline, 6, 12 & 18 months

			as determined with a baseline cardiopulmonary exercise test).<br>Setting: The supervised setting of a health care exercise facility.<br>Deliverer: Trained exercise professionals.<br>Involvement of family: NR<br>Other: HEART camp was based on Bandura's cognitive-behavioral strategies and provided a multicomponent approach of group-based and individual-based intervention delivery. The intervention included fitness testing, exercise prescriptions, supervised exercise, goal setting, monitoring and relapse prevention, etc.		
Pozehl, et al., 2008 (United States) [11]	Single center (A HF clinic in the Midwestern United States)<br><br>N randomized total: *N*=23<br>16 (I) vs. 7 (C)<br>N analyzed total: *N*=21<br>15 (I) vs. 6 (C)<br><br>Attrition rate: 8.70% (I: 1/16; C: 1/7)	Mean age (SD), years:<br>66.3 (9.6) vs. 66.0 (12.6)<br>total: 66.2 (10.2)<br>Female, n (%):<br>2 (13.3%) vs. 0 (0.0%),<br>total: 2 (9.5%)<br>White, n (%):<br>NR<br>Mean BMI (SD), kg/m^2:<br>27.8 (5.9) vs. 24.6 (4.4)<br>total: 26.9 (5.6)<br>HF etiology, n (%):<br>Ischemic: 15 (71.4%)<br>Non-ischemic: 6 (28.6%)<br>NYHA class, n (%):<br>II: 8 (38.1%)<br>III: 11 (52.4%)<br>IV: 2 (9.5%)<br>Mean LVEF (SD), %:<br>27.9 (7.0) vs. 29.7 (8.7)<br>total: 28.4 (7.3)	Intervention category: HF exercise and resistance training<br>Content: Each exercise session lasted 60 min: (1) 5-minute warm-up phase; (2) 30-minute aerobic phase (treadmills, stationary bikes, rowers, and arm ergometers were utilized according to individual tolerance); (3) 20-minute strength/resistance training including light upper-body exercises (military press, biceps curl, and lateral deltoid raises) and lower-body exercises (knee extension, side hip raise, and hip extension). Wall push-ups, abdominal curl-ups, and/or pelvic tilts were also included; (3) 5-minute cool-down phase.<br>**[Peripheral muscle contracted]**<br>Frequency: 3 days of exercise training per week for 24 weeks.<br>Duration: 6 months.<br>Intensity: (1) Aerobic exercise at 60~85% maximum VO_2 obtained from the baseline cardiopulmonary exercise test and a rating of 12~14 of perceived exertion on the Borg scale. (2) Strength/resistance training with 1~10 lb hand and ankle weights.<br>Setting: A standard cardiac rehabilitation setting and the maintenance area of the rehabilitation setting. (The first 12 weeks of the intervention were completed in a standard cardiac rehabilitation setting associated with the hospital. Subjects were instructed to exercise on their own in the maintenance area of the rehabilitation setting for weeks 12 through 24).<br>Deliverer: NR	Usual care for HF, including standard pharmacologic treatment at the HF clinic.	(1) Dyspnea (the Dyspnea Index) /baseline, 3 & 6 months<br>(2) Fatigue (the Piper Fatigue Scale, PFS) /baseline, 3 & 6 months

			Involvement of family: NR<br>Other: Strategies from social learning theory (goal-setting, feedback and problem-solving guidance) were utilized with the intervention group to facilitate and improve adherence to the training program.		
Pozehl, et al., 2010 (United States) [12]	Single center<br>(A regional cardiology center in the Midwestern United States)<br><br>N randomized<br>total: *N*=42<br>22 (I) vs. 20 (C)<br>N analyzed<br>total: *N*=31<br>15 (I) vs. 16 (C)<br><br>Attrition rate:<br>0% (I: 0/22<br>C: 0/20)	Mean age (SD), years:<br>57.3 (12.3) vs. 63.0(15.1)<br>total: 59.9 (13.8)<br>Female, n (%):<br>10 (45.5%) vs. 8 (45.5%),<br>total: 18 (45.5%)<br>White, n (%):<br>NR<br>Mean BMI (SD), kg/m^2:<br>NR<br>HF etiology, n (%):<br>Ischemic: 21 (50.0%)<br>Nonischemic: 21 (50.0%)<br>NYHA class, n (%):<br>II: 23 (54.8%)<br>III: 19 (45.2%)<br>Mean LVEF (SD), %:<br>33.2 (6.6) vs. 32.3 (5.5),<br>total: 32.7 (6.1)	Intervention category: HF exercise and resistance training<br>Content: (1) Subjects participated in small group educational sessions with topics pertinent to HF. Group sessions were held in the same frequency and format (i.e., weekly for the first 3 weeks and biweekly for weeks 4~12). (2) Participants met weekly during weeks 1 to 3 and biweekly during weeks 4 to 12 in small group sessions with a physical therapist and a nurse to guide the HF exercise and resistance training. The principal investigator taught resistance training during the first 3 weeks using detailed pictures and guidelines. Receipt of the treatment was assessed during each group session through questioning and verification of participant understanding. The principal investigator or physical therapist observed enactment of skills on a regular basis during the first 3 weeks of the study and every 4 weeks during the remaining 9 weeks. (3) Participants, with the guidance of the physical therapist, established goals for exercise each week. Exercise performance data were compared with goals, and the physical therapist provided feedback on goals and exercise performance. (4) Problem-solving strategies for any perceived difficulties or barriers to exercise were addressed in the group sessions. (5) The exercise training component of the HF Exercise and Resistance Training Camp Intervention (HEART Camp) consisted of aerobic exercise 3 times per week and resistance training twice per week.<br>**[Peripheral muscle contracted]**<br>Frequency: 3 times per week and each exercise session lasted 60 minutes (i.e., 15 minutes warm up, 30 minutes exercise, 15 minutes cool down).<br>Duration: 3 months.<br>Intensity: Aerobic at 40%-70% heart rate reserve as determined with a baseline cardiopulmonary exercise test. Resistance training was based on the	Attention control group received (1) with an equivalent amount of time spent with the nurse for intervention group.	(1) Dyspnea (Dyspnea-Fatigue Index, DFI)<br>/baseline & 3 months

			acclimation method with 1 to 3 sets of 8 repetitions each for 8 to 10 exercises involving upper and lower body.<br>Setting: A hospital-based cardiac rehabilitation setting and a maintenance rehabilitation facility (The first 3 weeks of aerobic training took place in a hospital-based rehabilitation setting and participants had electrocardiographic monitoring. The remaining 9 weeks, participants exercised 3 times weekly according to their own schedules in a maintenance rehabilitation facility).<br>Deliverer: A physical therapist and a nurse.<br>Involvement of family: NR<br>Other: HEART camp was based on Bandura's cognitive-behavioral strategies and provided a multicomponent approach of group-based and individual-based intervention delivery. The intervention included fitness testing, exercise prescriptions, supervised exercise, goal setting, monitoring and relapse prevention, etc.		
Seo, et al., 2016 (United States) [13]	Three centers (Two academic medical centers in a midwestern urban area and one primary care specialty clinic in a rural community)<br><br>N randomized<br>total: *N*=36<br>18 (I) vs. 18 (C)<br>N analyzed<br>total: *N*=25<br>10 (I) vs. 15 (C)	Mean age (SD), years:<br>65.2 (11.34) vs. 66.6 (13.69)<br>total: 65.9 (12.4)<br>Female, n (%):<br>total: 11 (28.6%)<br>White, n (%):<br>total: 33 (94.3%)<br>Mean BMI (SD), kg/m^2:<br>29.8 (5.8) vs. 32.3 (6.6)<br>total: 31.1 (6.3)<br>HF etiology, n (%):<br>Ischemic: 19 (54.3%)<br>Dilated: 17 (45.7%)<br>NYHA class, n (%):<br>II: 18 (51.4%)	Intervention category: Diaphragmatic breathing training<br>Content: (1) During the orientation, patients in the intervention group received detailed face-to-face instructions as to how to carry out the diaphragmatic breathing training at home. (2) The intervention group received audio CDs to guide them through the breathing training, paced at six breaths per minute with the diaphragm. Each CD had voice-guided directions with soothing background music so that patients could breathe with differing amounts of instruction. (3) Follow-up calls at weeks 1, 2, 4, and 6 to assess how the patients were doing with the deep and slow breathing retraining, to help them problem-solve solutions to any barriers that might have arisen, implement the breathing exercise, and provide them with positive feedback and encouragement to continue with the intervention.<br>**[Respiratory muscle contracted]**<br>Frequency: Week 1: 5 minutes, twice a day, at least 5 days per week; week 2: 10 minutes, twice a day, at least 5 days per week; weeks 3~8: 15 minutes, twice a day, at least 5 days per week.	The attention control group also received four telephone calls from a member of the research team at the same time points as the intervention group. General health promotion topics were discussed.	Dyspnea measured in three ways:<br>(1) Dyspnea frequency and burden (A composite of item 7 and item 8 from the Kansas City Cardiomyopathy Questionnaire, KCCQ)<br>/baseline, 2 & 5 months<br>(2) Dyspnea with ADL (measured by 8 items, e.g., dyspnea with dressing, doing housework, etc.)<br>/baseline, 2 & 5 months<br>(3) Dyspnea with physical functioning (measured by 10 items, e.g., dyspnea with carrying an

	Attrition rate:<br>25.0% (I: 6/18 C: 3/18)	III: 16 (45.7%)<br>IV: 1 (5.6%)<br>Mean LVEF (SD), %:<br>35.2 (21.29) vs. 38.9 (15.8)<br>total: 37.1 (18.6)	Duration: 2 months with a follow-up at 5 months.<br>Intensity: NR<br>Setting: CD guided at patient's home.<br>Deliverer: Research staff.<br>Involvement of family: NR<br>Other: The intervention was guided by theoretical concepts from social cognitive theory and was designed to build self-efficacy to perform breathing exercise through successful performance of a behavior (mastery), vicarious experiences, verbal persuasion by research staff, and physiological responses to breathing practice.		object while climbing stairs, etc.) /baseline, 2 & 5 months
Wall, et al., 2010 (United States) [14]	Single center (An outpatient, home-based, multidisciplinary chronic)<br><br>N randomized<br>total: N=19<br>9 (I) vs. 10 (C)<br>N analyzed<br>total: N=19<br>9 (I) vs. 10 (C)<br><br>Attrition rate:<br>21.05% (I: 1/9; C: 3/10)	Mean age (SD), years:<br>69 (4.44) vs. 70 (4.05)<br>total: 69.74 (4.13)<br>Female, n (%):<br>3 (33.3%) vs. 5 (50%)<br>total: 8 (42.1%)<br>White, n (%):<br>19 (100%)<br>Mean BMI (SD), kg/m^2:<br>NR<br>HF etiology, n (%):<br>NR<br>Mean NYHA class (SD):<br>2 (0) vs. 2.13 (0.13)<br>total: 2.07 (0.11)<br>Mean LVEF (SD), %:<br>NR	Intervention category: Home-based exercise training<br>Content: Participants were enrolled in a home-based, multi-disciplinary chronic disease management program, which included multiple home visits and follow-up phone calls.<br>In addition to receiving a comprehensive disease management program, the intervention group participated in a supervised home-based exercise program. (1) Subjects were provided with Lifestyler® treadmills for use in their home free of charge. (2) Subjects received three supervised exercise sessions with EKG monitoring at Griffin Hospital with cardiac rehabilitation specialist. (3) Subjects received weekly in-home exercise visits with cardiac rehabilitation specialist for month 1 and monthly in-home exercise visits with cardiac rehabilitation specialist for months 2~12. (4) The exercise regimen consisted of treadmill use for at least 15 minutes, 3 times per week at varied grades, speeds, and durations depending on symptom severity.<br>**[Peripheral muscle contracted]**<br>Frequency: 3 times per week.<br>Duration: 12 months.<br>Intensity: The exercise intensity at varied grades, speeds, and durations depending on symptom severity.<br>Setting: Home.	The control group only received home-based, multi-disciplinary chronic disease management program, including 3-4 in-home visits from a case manager in month 1, bi-monthly phone calls in months 2-7, a group education session, and monthly phone calls in months 8-12.	(1) Dyspnea (Chronic Heart Failure Questionnaire, CHQ subscale) /baseline, 6 & 12 months<br>(2) Fatigue (Chronic Heart Failure Questionnaire, CHQ subscale) /baseline, 6 & 12 months

			Deliverer: A dedicated cardiac rehabilitation specialist.<br>Involvement of family: NR<br>Other: N/A		
Weiner, et al.,<br>1999<br>(Israel) [15]	Single center<br>(Department of Medicine A and Cardiology, Hillel-Yaffe Medical Center in Hadera, Israel)<br><br>N randomized<br>total: N=20<br>10 (I) vs. 10 (C)<br>N analyzed<br>total: *N*=20<br>10 (I) vs. 10(C)<br><br>Attrition rate:<br>NR	Mean age (SD), years:<br>66.2 (4.6) vs. 63.8 (4.0)<br>total: 65 (4.4)<br>Female, n (%):<br>2 (10%)<br>White, n (%):<br>NR<br>Mean BMI (SD), kg/m^2:<br>NR<br>HF etiology, n (%):<br>NR<br>NYHA class, n (%):<br>2.3 (0.2) vs. 2.4 (0.2)<br>total: 2.35 (0.2)<br>Mean LVEF (SD), %:<br>24.7 (1.6) vs. 22.9 (2.4)<br>total: 23.8 (2.2)	Intervention category: Inspiratory muscle training<br>Content: (1) Subjects in the treatment group received specific inspiratory muscle training with a threshold inspiratory muscle trainer. (2) The subjects started breathing at a resistance equal to 15% of their PImax for 1 week. The resistance was then increased incrementally, 5% each session, to reach 60% of their PImax at the end of the 1st month. (3) The training was then continued for the next 2 months at 60% of the subjects' PImax, adjusted every week to the new PImax achieved.<br>**[Respiratory muscle contracted]**<br>Frequency: One hour daily, 6 times/week, for 30 minutes, for 3 months.<br>Duration: 3 months.<br>Intensity: Resistance was 15% of their PImax for 1 week and then increased incrementally, 5% each session, to 60% by the end of 1 month. The training continued on 60% for the next 2 months and was adjusted based on the PImax achieved.<br>Setting: Department of Medicine A and Cardiology.<br>Deliverer: A physiotherapist.<br>Involvement of family: NR<br>Other: N/A	Patients in the control group received sham training with the same device, but with no resistance.	(1) Dyspnea (Dyspnea Index)<br>/baseline & 3 months
Willenheimer, et al., 1998 & 2001<br>(Sweden)<br>[16,17]	Single center<br>(Malmö University Hospital)<br><br>N randomized<br>total: N=50	Mean age (SD), years:<br>64 (5) vs. 64 (9)<br>total: 64 (7.4)<br>Female, n (%):<br>6 (27%) vs. 8 (30%),<br>total: 14 (29%)<br>White, n (%):	Intervention category: Exercise training<br>Content: (1) The treatment group performed interval training on a cycle ergometer: 90s exercise and 30s rest. Patients individually adjusted the speed and load in order to reach the adequate intensity. The training was performed as a group, supervised by a physiotherapist who ensured that the protocol was complied with. (2) The 4-month active study period was followed by a 6-month extended follow-up. Neither training patients nor controls were	The control patients agreed not to change their degree of physical activity during the study and did not participate in group exercise	(1) Dyspnea (Dyspnea-Fatigue Index, DFI)<br>/baseline, 4 & 10 months

	23 (I) vs. 27 (C)<br>N analyzed<br>total: *N*=37<br>17 (I) vs. 20 (C)<br><br>Attrition rate:<br>26.0% (I: 6/23; C: 7/27)	NR<br>Mean BMI (SD), kg/m^2:<br>NR<br>HF etiology, n (%):<br>Ischemic: 37 (75.5%)<br>Nonischemic: 12 (24.5%)<br>NYHA class, n (%):<br>I: 6 (12.2%)<br>II: 19 (38.8%)<br>III: 24 (49.0%)<br>Mean LVEF (SD), %:<br>35 (11) vs. 36 (11)<br>total: 35.6 (10.9)	instructed regarding physical activity during the 6-month extended follow-up.<br>**[Peripheral muscle contracted]**<br>Frequency: The exercise time was gradually increased from 15 min twice a week to 45 min three times a week from week 7, for a total of 16 weeks.<br>Duration: 4 months with a follow-up at 10 months.<br>Intensity: Patients in sinus rhythm exercised at a digitally displayed heart rate corresponding to 80% of VO_2-max (at the baseline exercise test) ± 5 beats·min^{-1}, for as long as possible during each interval. In patients with atrial fibrillation, exercise intensity corresponded to exhaustion grade 15, according to the Borg scale (approximately 80% of maximum).<br>Setting: The training was performed as a group supervised by a physiotherapist.<br>Deliverer: A physiotherapist.<br>Involvement of family: NR<br>Other: N/A	training.	
Yu, et al., 2007 (Hong Kong, China) [18]	One center (Medical unit of a university-affiliated hospital, The Chinese University of Hong Kong, Hong Kong, PR China)<br><br>N randomized<br>total: N=158<br>79 (I) vs. 79 (C)<br>N analyzed<br>total: N=121<br>59 (I) vs. 62 (C)	Mean age (SD), years:<br>74.9 (8.0) vs. 77.4 (7.5)<br>total: 76.2 (7.8)<br>Female, n (%):<br>26 (44.1%) vs 35 (56.5%)<br>total: 61 (50.4%)<br>White, n (%):<br>NR<br>Mean BMI (SD), kg/m2:<br>NR<br>HF etiology, n (%):<br>NR<br>NYHA class, n (%):<br>I: 4 (3.3%)<br>II: 63 (52.1%)	Intervention category: Progressive muscle relaxation training<br>Content: (1) The intervention group attended a Grade 2 progressive muscle relaxation training (PMRT) program including two 1-hr PMRT sessions every week after their hospital discharge as well as one revision workshop. The training involved systematically tensing and relaxing 16 muscle groups with a regular breathing pattern to enhance relaxation. In the first session, a nurse taught the training process, and in the second session, the participants demonstrated the relaxation technique using the taped instructions of the nurse's voice. (2) Participants took the audiocassette tapes with the same instructions home with them to practice twice daily throughout the study period. (3) They were asked to promptly record their relaxation practices in a practice log. (4)A skill revision workshop was held 4 weeks later to reassess the participants' skill mastery and to discuss their concerns about the muscle relaxation training home practice. (5) The interventionist initiated biweekly telephone calls to encourage the participants' compliance and clarify related	Patients in the control group received two weekly telephone calls followed by biweekly for the 14-week study period. The inertness of the attention control intervention was maintained by limiting the conversation to a general greeting.	(1) Dyspnea (The dyspnea subscale of the Chronic Heart Failure Questionnaire, CHQ subscale) /baseline, 2 & 3.5 months.<br>(2) Fatigue (The fatigue subscale of the Chronic Heart Failure Questionnaire, CHQ subscale) /baseline, 2 & 3.5 months.

	Attrition rate: 23.42% (I: 20/79; C: 17/79)	III: 42 (34.7%)<br>IV: 3 (2.5%)<br>Mean LVEF (SD), %:<br>NR	problems. A telephone record form was developed to record the topics discussed.<br>**[Peripheral muscle contracted]**<br>Frequency: Two 1-hr PMRT sessions every week for 14 weeks.<br>Duration: 3.5 months.<br>Intensity: Tensing the muscle to three fourths of the full possible tension for a shorter period of 5 seconds.<br>Setting: Hospital and home.<br>Deliverer: A research nurse.<br>Involvement of family: NR<br>Other: Patients in both the groups received the same usual postdischarge care consisting of medication instructions and regular medical follow-up at the outpatient clinic.		

† Intervention (I) versus Control (C) unless otherwise specified.

Note: HF, heart failure; SD, standard deviation; NYHA Class, New York Heart Association (NYHA) functional classification; LVEF, left ventricular ejection fraction; IQR, the interquartile range; BMI, body mass index.

References to studies included in this review

1. Alkan, H., Uysal, H., Enç, N., & Yigit, Z. (2017). Influence of breathing exercise education applied on patients with heart failure on dyspnoea and quality of sleep: A randomized controlled study. *International Journal of Medical Research & Health Sciences*, 6(9), 107-113.
2. Beniaminovitz, A., Lang, C. C., LaManca, J., & Mancini, D. M. (2002). Selective low-level leg muscle training alleviates dyspnea in patients with heart failure. *Journal of the American College of Cardiology*, 40(9), 1602-1608. doi:10.1016/s0735-1097(02)02342-2
3. Bosnak-Guclu, M., Arikan, H., Savci, S., Inal-Ince, D., Tulumen, E., Aytemir, K., & Tokgözoglu, L. (2011). Effects of inspiratory muscle training in patients with heart failure. *Respiratory Medicine*, 105(11), 1671-1681. doi:10.1016/j.rmed.2011.05.001
4. Chen, D. M., Yu, W. C., Hung, H. F., Tsai, J. C., Wu, H. Y., & Chiou, A. F. (2018). The effects of Baduanjin exercise on fatigue and quality of life in patients with heart failure: A randomized controlled trial. *European journal of cardiovascular nursing*, 17(5), 456-466. doi: 10.1177/1474515117744770
5. Corvera-Tindel, T., Doering, L. V., Woo, M. A., Khan, S., & Dracup, K. (2004). Effects of a home walking exercise program on functional status and symptoms in heart failure. *American Heart Journal*, 147(2), 339-346. doi: 10.1016/j.ahj.2003.09.007
6. Hossein Pour, A. H., Gholami, M., Saki, M., & Birjandi, M. (2020). The effect of inspiratory muscle training on fatigue and dyspnea in patients with heart failure: A randomized, controlled trial. *Japan Journal of Nursing Science*, 17(2), e12290. doi: 10.1111/jjns.12290
7. Jena, S., Das, S., & Pradhan, R. (2020). A comparative study between effects of aerobic exercises and conventional treatment on selected outcomes of heart failure clients. *International Journal of Research in Pharmaceutical Sciences*, 11(1), 342-346.
8. Jin, H, & Lee, H. (2016). The effects of reinforced walking exercise on dyspnea-fatigue symptoms, daily activities, walking ability, and health related quality of life in heart failure patients. *Korean Journal of Adult Nursing*, 28(3), 266-278. doi:10.7475/kjan.2016.28.3.266.
9. Klocek, M., Kubinyi, A., Bacior, B., & Kawecka-Jaszcz, K. (2005). Effect of physical training on quality of life and oxygen consumption in patients with congestive heart failure. *International Journal of Cardiology*, 103(3), 323-329. doi: 10.1016/j.ijcard.2004.10.021
10. Norman, J. F., Kupzyk, K. A., Artinian, N. T., Keteyian, S. J., Alonso, W. S., Bills, S. E., & Pozehl, B. J. (2020). The influence of the HEART Camp intervention on physical function, health-related quality of life, depression, anxiety and fatigue in patients with heart failure. *European journal of cardiovascular nursing*, 19(1), 64-73. doi: 10.1177/1474515119867444
11. Pozehl, B., Duncan, K., & Hertzog, M. (2008). The effects of exercise training on fatigue and dyspnea in heart failure. *European journal of cardiovascular nursing*, 7(2), 127-132. doi: 10.1016/j.ejcnurse.2007.08.002
12. Pozehl, B., Duncan, K., Hertzog, M., & Norman, J. F. (2010). Heart Failure Exercise And Training Camp: effects of a multicomponent exercise training intervention in patients with heart failure. *Heart & lung*, 39(6 Suppl), S1-S13. doi: 10.1016/j.hrtlng.2010.04.008
13. Seo, Y., Yates, B., LaFramboise, L., Pozehl, B., Norman, J. F., & Hertzog, M. (2016). A home-based diaphragmatic breathing retraining in rural patients with heart failure. *Western Journal of Nursing Research*, 38(3), 270-291. doi: 10.1177/0193945915584201
14. Wall, H. K., Ballard, J., Troped, P., Njike, V. Y., & Katz, D. L. (2010). Impact of home-based, supervised exercise on congestive heart failure. *International Journal of Cardiology*, 145(2), 267-270. doi: 10.1016/j.ijcard.2009.09.478

15. Weiner, P., Waizman, J., Magadle, R., Berar-Yanay, N., & Pelled, B. (1999). The effect of specific inspiratory muscle training on the sensation of dyspnea and exercise tolerance in patients with congestive heart failure. *Clinical Cardiology*, 22(11), 727-732. doi: 10.1002/clc.4960221110
16. Willenheimer, R., Erhardt, L., Cline, C., Rydberg, E., & Israelsson, B. (1998). Exercise training in heart failure improves quality of life and exercise capacity. *European Heart Journal*, 19(5), 774-781. doi: 10.1053/euhj.1997.0853
17. Willenheimer, R., Rydberg, E., Cline, C., Broms, K., Hillberger, B., Oberg, L., & Erhardt, L. (2001). Effects on quality of life, symptoms and daily activity 6 months after termination of an exercise training programme in heart failure patients. *International Journal of Cardiology*, 77(1), 25-31. doi: 10.1016/s0167-5273(00)00383-1
18. Yu, D. S., Lee, D. T., Woo, J. (2007). Effects of relaxation therapy on psychologic distress and symptom status in older Chinese patients with heart failure. *Journal of Psychosomatic Research*, 62(4), 427-437. doi: 10.1016/j.jpsychores.2006.10.012

Supplementary Figure S1: Preliminary meta-analysis for dyspnea

	Exercise			Control				Std. Mean Difference
**Study or Subgroup**	**Mean**	**SD**	**Total**	**Mean**	**SD**	**Total**	**Weight**	**IV, Random, 95% CI**
Alkan 2017	-2.2	1.29	29	-0.66	2.07	27	9.6%	-0.89 [-1.44, -0.34]
Beniaminovitz 2002	-3.7	0.5	17	-1.3	0.8	8	7.9%	-3.82 [-5.25, -2.39]
Bosnak-Guclu 2011	-1.2	0.84	16	-0.22	0.88	14	9.3%	-1.11 [-1.89, -0.33]
Hossein Pour 2020	-1.25	0.21	42	0.09	0.03	42	7.8%	-8.85 [-10.29, -7.42]
Jin 2016	-2.88	1.8	16	-2.12	1.57	25	9.5%	-0.45 [-1.08, 0.19]
Pozehl 2008	-1.08	1.8	15	0.33	2.66	6	8.9%	-0.66 [-1.63, 0.32]
Pozehl 2010	-1.4	2.31	15	0.3	2.59	16	9.4%	-0.67 [-1.40, 0.05]
Seo 2016	-0.65	0.51	10	-0.64	0.42	15	9.2%	-0.02 [-0.82, 0.78]
Wall 2010	0.44	1.41	9	-0.5	1.3	10	9.0%	0.66 [-0.27, 1.59]
Willenheimer 1998	-0.5	1.93	17	-0.2	1.85	20	9.5%	-0.16 [-0.80, 0.49]
Yu 2007	-1.1	0.93	59	-0.58	0.93	62	9.9%	-0.56 [-0.92, -0.19]
**Total (95% CI)**			**245**			**245**	**100.0%**	**-1.36 [-2.22, -0.50]**

Heterogeneity: $Tau^2 = 1.91$; $Chi^2 = 158.40$, df = 10 ($P < 0.00001$); $I^2 = 94\%$
Test for overall effect: $Z = 3.10$ ($P = 0.002$)

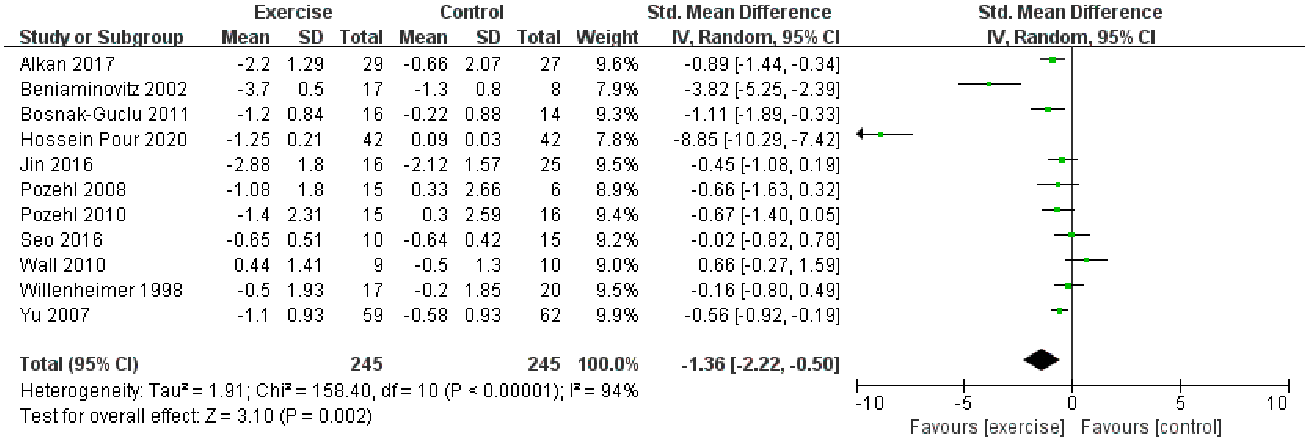

Supplementary Figure S2: Preliminary meta-analysis for fatigue

	Exercise			Control				Std. Mean Difference
**Study or Subgroup**	**Mean**	**SD**	**Total**	**Mean**	**SD**	**Total**	**Weight**	**IV, Random, 95% CI**
Bosnak-Guclu 2011	-15.64	13	16	-9.93	14.54	14	14.0%	-0.40 [-1.13, 0.32]
Chen 2018	-3.4	7.7	30	2.21	7.3	33	15.0%	-0.74 [-1.25, -0.23]
Hossein Pour 2020	-14.41	6.35	42	0.83	2.19	42	14.4%	-3.18 [-3.83, -2.53]
Norman 2020	-2.4	10	71	0.6	9.95	88	15.7%	-0.30 [-0.61, 0.02]
Pozehl 2008	-1.08	2.18	15	-1.09	2.07	6	12.8%	0.00 [-0.94, 0.95]
Wall 2010	0.22	1.02	9	-0.9	0.94	10	12.6%	1.09 [0.11, 2.07]
Yu 2007	-0.63	1.03	59	-0.2	1.12	62	15.6%	-0.40 [-0.76, -0.04]
**Total (95% CI)**			**242**			**255**	**100.0%**	**-0.59 [-1.33, 0.15]**

Heterogeneity: $Tau^2 = 0.88$; $Chi^2 = 78.28$, df = 6 ($P < 0.00001$); $I^2 = 92\%$
Test for overall effect: $Z = 1.58$ ($P = 0.12$)

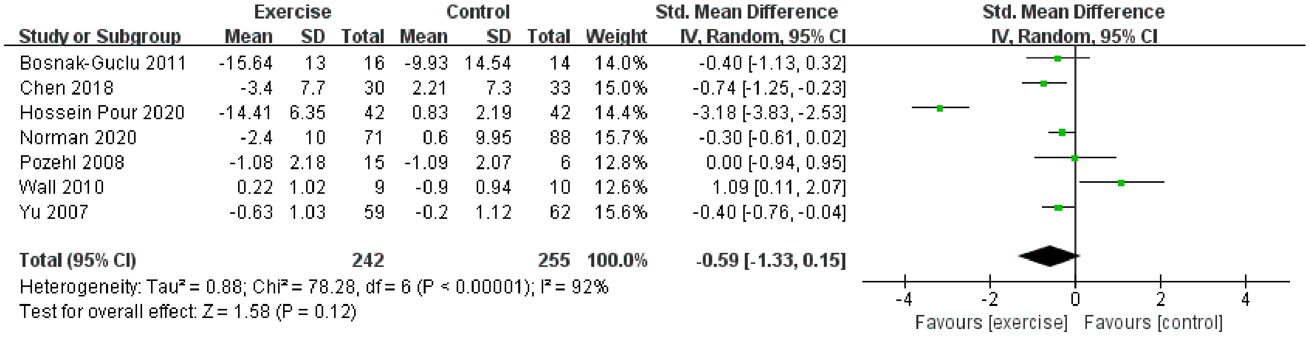

Supplement: Supplementary file 1 [file biomedicines-10-01111-s001.zip › biomedicines-1717563-supplementary.pdf]
